# Arabidopsis PCH2 Mediates Meiotic Chromosome Remodeling and Maturation of Crossovers

**DOI:** 10.1371/journal.pgen.1005372

**Published:** 2015-07-16

**Authors:** Christophe Lambing, Kim Osman, Komsun Nuntasoontorn, Allan West, James D. Higgins, Gregory P. Copenhaver, Jianhua Yang, Susan J. Armstrong, Karl Mechtler, Elisabeth Roitinger, F. Chris H. Franklin

**Affiliations:** 1 School of Biosciences, University of Birmingham, Edgbaston, Birmingham, United Kingdom; 2 Department of Biology and Carolina Center for Genome Scientists, University of North Carolina, Chapel Hill, Chapel Hill, North Carolina, United States of America; 3 IMP-IMBA, Vienna, Austria; Karlsruhe Institute of Technology, GERMANY

## Abstract

Meiotic chromosomes are organized into linear looped chromatin arrays by a protein axis localized along the loop-bases. Programmed remodelling of the axis occurs during prophase I of meiosis. Structured illumination microscopy (SIM) has revealed dynamic changes in the chromosome axis in *Arabidopsis thaliana* and *Brassica oleracea*. We show that the axis associated protein ASY1 is depleted during zygotene concomitant with synaptonemal complex (SC) formation. Study of an *Atpch2* mutant demonstrates this requires the conserved AAA+ ATPase, PCH2, which localizes to the sites of axis remodelling. Loss of PCH2 leads to a failure to deplete ASY1 from the axes and compromizes SC polymerisation. Immunolocalization of recombination proteins in *Atpch2* indicates that recombination initiation and CO designation during early prophase I occur normally. Evidence suggests that CO interference is initially functional in the mutant but there is a defect in CO maturation following designation. This leads to a reduction in COs and a failure to form COs between some homologous chromosome pairs leading to univalent chromosomes at metaphase I. Genetic analysis reveals that CO distribution is also affected in some chromosome regions. Together these data indicate that the axis remodelling defect in *Atpch2* disrupts normal patterned formation of COs.

## Author Summary

In the reproductive cells of many eukaryotes, a process called meiosis generates haploid gametes. During meiosis, homologous parental chromosomes (homologs) recombine forming crossovers (CO) that provide genetic variation. CO formation generates physical links called chiasmata, which are essential for accurate homolog segregation. CO control designates a sub-set of recombination precursors that will mature to form at least one chiasma between each homolog pair. Recombination is accompanied by extensive chromosome reorganization. Formation of a proteinaceous axis organizes the pairs of sister chromatids of each homolog into conjoined linear looped chromatin arrays. Pairs of homologs then align and synapse becoming closely associated along their length by a protein structure, the synaptonemal complex (SC). The SC is disassembled at the end of prophase I and recombination is completed. We have investigated the link between recombination and chromosome remodelling by analysing the role of a protein, PCH2, which we show is required for remodelling of the chromosome axis during SC formation. In wild type, immunolocalization reveals depletion of the axis-associated signal of the axis component, ASY1, along synapsed regions of the chromosomes. In the absence of PCH2, the ASY1 signal is not depleted from the chromosome axis and the SC does not form normally. Although this defect in chromosome remodelling has no obvious effect on CO designation, CO maturation is perturbed such that the formation of at least one CO per homolog pair no longer occurs.

## Introduction

During meiosis genetic crossovers (COs), the products of homologous recombination, in conjunction with sister chromatid cohesion establish physical links, referred to cytologically as chiasmata, between homologous chromosome pairs (homologs) to ensure accurate chromosome segregation at the first nuclear division that follows prophase I. In the absence of crossing over the homologs segregate randomly. This leads to the formation of aneuploid gametes following the second meiotic division [1]. Recombination is initiated by the programmed formation of DNA double-strand breaks (DSBs), catalysed by the topoisomerase type II related protein Spo11 [2,3]. In *Saccharomyces cerevisiae* (budding yeast) around 40% of DSBs are repaired as non-CO (NCO) products with the remainder progressing to form COs [4]. In *Arabidopsis thaliana* and other multicellular organisms the proportion of COs is substantially less, typically 5–10% [5]. Importantly, the CO/NCO balance is highly controlled. This control is manifested in several ways. First, each pair of homologs acquires at least one CO. Second, CO interference ensures that multiple COs are well spaced along the chromosomes. Finally, CO homeostasis maintains CO numbers in the face of perturbations that may affect the number of earlier recombinational interactions [6–10]. It is hypothesized that a CO patterning phenomenon, that can be simulated by the beam-film model, underlies these three features of CO control [11,12].

In budding yeast, DSBs form in early leptotene coincident with the elaboration of a proteinaceous chromosome axis that organizes each pair of sister chromatids into linear looped chromatin arrays conjoined by a shared axis. DSBs occur in the context of the chromosome axis [13–15]. At the transition from leptotene to zygotene, formation of the synaptonemal complex (SC), a tripartite structure comprising the chromosome axes linked by overlapping transverse filaments (TFs), is initiated at multiple synapsis initiation sites [1,16,17]. Synapsis continues throughout zygotene bringing the axes into close apposition and is completed at the onset of pachytene when the SC is fully formed. This programmed morphogenesis of the chromosome axes and SC is critical for the coordination of recombination, playing important roles in the meiosis-specific bias that favours inter-homolog recombination and the maturation of CO designated recombination intermediates [18–26].

In budding yeast mutation of the *PCH2* gene, which encodes a member of the conserved AAA+ ATPase protein family, disrupts remodelling of the chromosome axis during prophase I of meiosis [27,28]. In wild type cells the chromosome axis protein, Hop1, and the SC transverse filament protein, Zip1, appear to load uniformly at a basal level along the chromosomes. Superimposed on this, each forms a series of non-overlapping, alternating hyper-abundant domains. In a *pch2* mutant this domainal loading is disrupted to give a uniform overlapping signal for each protein along the chromosomes [27,28]. Pch2 may modulate inter-homolog bias by remodelling the chromosome structure in the vicinity of DSBs and have a role in a recombination checkpoint [29,30]. Loss of the protein also affects CO formation. In one study, a *pch2∆* mutant had increased COs on larger chromosomes, while CO frequency on the small chromosome III was unaffected [31]. Genetic data suggested the mutant also exhibited a defect in CO interference. A link with CO interference was also established in a parallel study, although in this instance no effect on overall CO number was observed [28]. However, further analysis based on the distribution of foci of the E3 ligase Zip3, which arise at CO designated intermediates and so provide an early marker for CO interference, reported that interference is not affected in a *pch2* deletion mutant [32].

Orthologs of *PCH2* have been identified in a variety of organisms. In mouse, analysis of a weak hypomorphic allele of *TRIP13* (*PCH2*) indicated that the protein was required for the efficient repair of DSBs that enter the NCO pathway but not COdesignated intermediates, which were processed normally. Despite the presence of unrepaired DSBs synapsis was normal in these mice [33]. Subsequently, a study of a more severe *Trip13* mutant reported a defect in CO formation and synapsis [34]. Similar to Pch2 in budding yeast, TRIP13 is required for the depletion of the Hop1 orthologs HORMAD1 and HORMAD2 along synapsed regions of the chromosome axes [35]. In Drosophila, PCH2 acts in a checkpoint to monitor defects in recombination and chromosome structure [36]. In *Caenorhabditis elegans* it is reported to maintain the fidelity of recombination and synapsis during prophase I by acting to constrain these processes [37]. A *PCH2* ortholog, referred to as *CRC1 (CENTRAL REGION COMPONENT1)* has also been identified in rice (*Oryza sativa*) [38]. The CRC1 protein is 43.8% identical to TRIP13 and 23.1% identical to Pch2 from budding yeast. The *crc1* mutant is completely asynaptic and forms univalents at metaphase I due to a failure to make DSBs [39].

Here we describe the identification and analysis of the PCH2 orthologs from *Brassica oleracea* and its close relative *Arabidopsis thaliana*. Using super-resolution structured illumination microscopy (SIM) we reveal dynamic changes in localization of PCH2 in relation to chromosome axis and SC morphogenesis during meiotic prophase I. Analysis of Arabidopsis mutants lacking PCH2 reveals a meiotic role that is markedly different to that reported for the rice CRC1 protein. Loss of PCH2 results in a failure to deplete ASY1 from the chromosome axes during zygotene coupled with a synaptic defect. Although recombination initiation and CO designation appears to occur normally during early prophase I, the defects in remodelling of the chromosome axes which influence SC formation are associated with a disruption of the patterned formation of COs along the homologous chromosomes.

## Results

### PCH2 and ASY1 co-immunoprecipitate in a meiotic protein complex

Protein complexes were immunoprecipitated from *Brassica oleracea* var. alboglabra A12DH pollen mother cells (PMCs) in meiotic prophase I using an anti-ASY1 antibody as previously described [40]. Co-precipitating proteins were analysed by mass-spectrometry and identified using the *Brassica rapa* sequence [41]. Up to 10 unique peptides corresponding to 25% sequence coverage (124/490 amino acids) of the Bra013827 predicted gene product were detected in three independent experiments and were absent from control samples (S1A Fig). The protein was identified as a P-loop containing nucleoside triphosphate hydrolase superfamily member. BLAST searches revealed that the protein is 87% identical to the Arabidopsis At4g24710 predicted gene product. ClustalW2 analysis (http://www.ebi.ac.uk) showed that Bra013827 and At4g24710 are members of a sub-family of the AAA+ ATPase super-family that contains the budding yeast *PCH2* and mouse *TRIP13* genes (S1B Fig).

### *Atpch2* mutants exhibit a reduced fertility phenotype

To determine whether At4g24710 encodes a functional ortholog of Pch2/TRIP13 we obtained three T-DNA insertion lines: SAIL_1187_C06, SALK_031449 and SALK_130138, hereafter referred to as *Atpch2-1*, *Atpch2-2* and *Atpch2-3* respectively. For all lines, the T-DNA insertion site was confirmed by DNA sequencing and the absence of a full-length *AtPCH2* transcript confirmed by RT-PCR (S2 and S3 Figs). The vegetative phenotype of each line was indistinguishable from wild type Arabidopsis, Col-0, but their fertility was reduced (S4A and S4B Fig). Quantification of the fertility defect in *Atpch2-1* revealed a slight, yet significant reduction in mean silique length from 1.66 ± 0.05 cm in wild type to 1.41 ± 0.06 cm (n = 50; P<0.05) in *Atpch2-1* (n = 50). This was accompanied by numerous gaps between the seeds within the siliques such that overall the mean seed-set was significantly reduced from 67.8 per silique in wild type to 34.6 in *Atpch2-1* (n = 50; P< 0.01). Analysis of *Atpch2-2* and *Atpch2-3* revealed very similar fertility defects (S4C Fig).

### Loss of AtPCH2 results in a reduction of chiasmata

**Fig 1:**
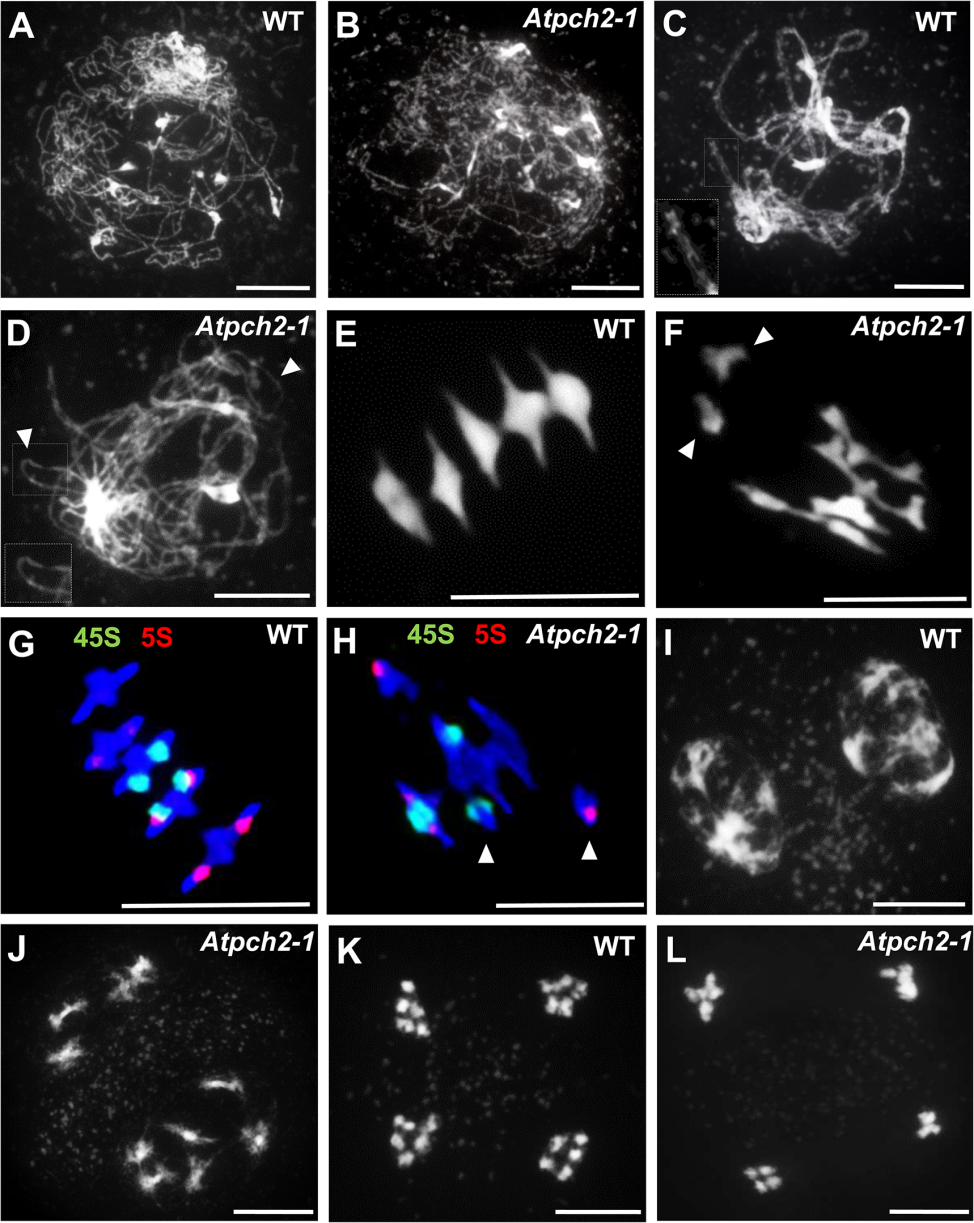
Meiotic stages from wild type Arabidopsis and *Atpch2-1* pollen mother cells. Chromosome spread preparations from wild type **(A,C,E,G,I,K)** and *Atpch2-1* **(B,D,F,H,J,L)** PMCs. **(A,B)** leptotene; **(C,D)** pachytene (note in *Atpch2-1* cell is at late prophase I normal pachytene stage was not observed). Arrowheads mark unsynapsed regions. **(E,F)** metaphase I. Arrowheads mark univalent chromosomes; **(G,H)** metaphase I stage labelled with 5S (red) and 45S (green) rDNA probes to identify the individual chromosomes. Arrowheads mark univalent chromosomes; **(I,J)** dyad; **(K-L)** tetrad. DNA is stained with DAPI. Bar = 10 μm.

The *Atpch2* reduced fertility phenotype suggested a meiotic defect. Cytogenetic analysis of DAPI stained chromosome spreads from *Atpch2-1* PMCs at leptotene revealed the threadlike chromosomes with no obvious differences to the wild type controls (Fig 1A and 1B). In wild type PMCs, the homologs achieved full synapsis at pachytene with the threadlike signals visibly paired along their lengths, giving a thicker appearance than at leptotene (Fig 1C). However, in *Atpch2-1* pachytene stage cells were not observed, instead the majority of the chromosomes remained as single threadlike signals with some limited regions where paired axes were visible (Fig 1D). During diplotene both *Atpch2-1* and wild type chromosomes desynapsed and began to condense, such that by diakinesis chiasmata linking the homologs were visible. At metaphase I, following further condensation, distinct bivalents were observed. Five bivalents were invariably present in wild type, but some *Atpch2-1* nuclei contained a mixture of bivalent and univalent chromosomes (Fig 1E and 1F). To quantify this we counted chiasmata in *Atpch2-1* in metaphase I chromosome spreads using fluorescent *in situ* hybridization (FISH) with 45S and 5S rDNA probes to identify individual chromosomes [42] (Fig 1G and 1H). This revealed a significant reduction in the mean chiasma frequency in *Atpch2-1* compared to wild type (6.9; n = 50 versus 9.6; n = 50; P < 0.001). No univalents were observed in the wild type sample, whereas they were present at a frequency of 10.0% in *Atpch2-1* with all chromosomes affected. Similar results were obtained for *Atpch2-2* (6.9; n = 37; P < 0.001; univalent frequency 14.6%) and *Atpch2-3* (6.2; n = 26; P < 0.001; univalent frequency 7.7%). As a consequence of this, in contrast to wild type, mis-segregation of the chromosomes was observed at the first meiotic division in *Atpch2-1* (Fig 1I and 1J) leading to unbalanced tetrads (Fig 1K and 1L). No precocious sister chromatid separation was observed suggesting that there was no cohesion defect. Analyses of *Atpch2-2* and *Atpch2-3* revealed that the meiotic defect in the three mutants is essentially identical (S5A–S5H Fig). To confirm that the observed phenotype was due to a loss of AtPCH2 function, an allelism test was conducted by crossing *Atpch2-1* with *Atpch2-2*. Phenotypic and cytological analysis of the *Atpch2-1/Atpch2-2* progeny revealed the same defects as in the parental lines confirming that these arose due to the loss of AtPCH2 function (S5I–S5M Fig).

We next investigated if the reduction in chiasmata in *Atpch2-1* reflected a defect in CO formation. Approximately 85% of COs in Arabidopsis exhibit CO interference [43]. Formation of these, so-called Class I COs, require a group of proteins known as ZMMs (Zip1, Zip2, Zip3/Hei10, Zip4, Mer3, Msh4 and Msh5) [5,18]. The remainder (Class II) are insensitive to CO interference and dependent on the structure-specific endonuclease AtMUS81 [44]. To determine if the loss of AtPCH2 affected one or both classes of COs we generated an *Atmsh5-1/Atpch2-1* double mutant. In *Atmsh5-1*, the number of chiasmata per PMC ranged between 0 and 4 with a mean chiasma frequency of 1.2 (n = 50) (S6B Fig). In comparison, the mean chiasma frequency in the *Atmsh5-1/Atpch2-1* double mutant was significantly reduced to 0.3 (n = 50; P < 0.001), with the number of chiasmata per nucleus ranging between 0 and 2 (S6C Fig).

Thus, overall the cytological analysis suggests that the reduction in chiasmata in *Atpch2-1* arises from a recombination defect that impacts on both interference sensitive and insensitive CO formation, rather than through an effect on sister chromatid cohesion.

### Chromosome axis remodelling is disrupted in *Atpch2-1*

**Fig 2:**
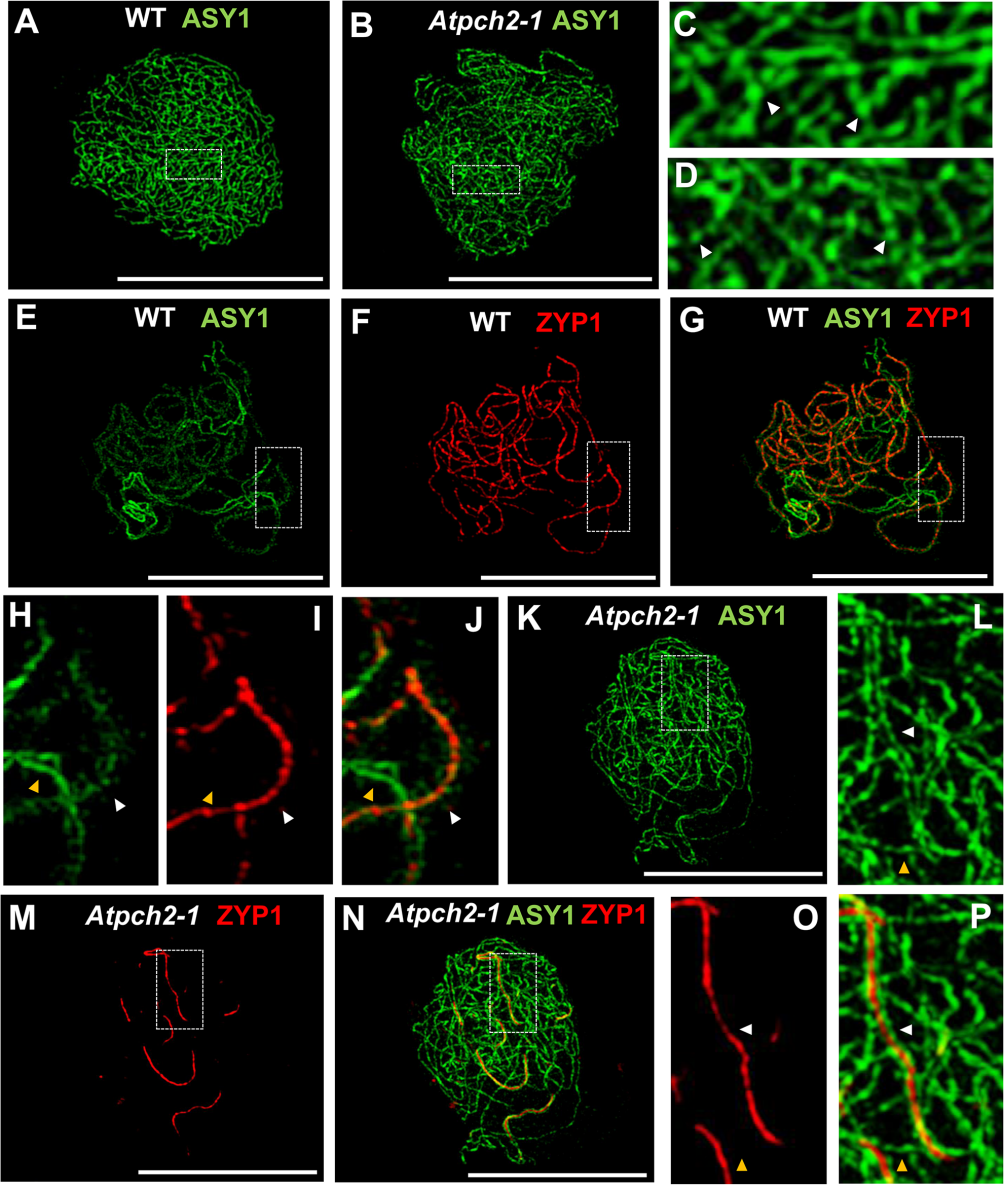
Immunolocalization of ASY1 and ZYP1 in wild type and *Atpch2-1* during prophase I. **(A-D)** Immunolocalization of ASY1 (green) on chromosome spread preparations from wild type **(A,C)** and *Atpch2-1* **(B,D)** nuclei at leptotene. Panels **C** and **D** show magnified sections of axes from **A** and **B** respectively. White arrowheads mark the regions of ASY1 with higher signal intensity. **(E-J)** Immunolocalization of ASY1 (green) **(E,G,H,J)** and ZYP1 (red) **(F,G,I,J)** and merge images **(G,J)** on chromosome spreads from wild type at zygotene. Figs **H, I** and **J** show magnified sections of axes from **E, F** and **G** respectively. **(K-P)** Immunolocalization of ASY1 (green) **(K,L,N,P)** and ZYP1 (red) **(M-P)** and merge images **(N,P)** on chromosome spread preparations from *Atpch2-1* at mid/late-prophase I. Figs **L, O** and **P** show magnified sections of axes from **K, M** and **N** respectively. White arrowheads represent synapsed regions while yellow arrowheads represent unsynapsed regions. DNA is stained with DAPI (blue). Bar = 10 cm.

The failure to observe pachytene stage PMCs in *Atpch2-1* suggested a defect in formation of the SC. To investigate further, we examined chromosome axis reorganization during early to mid-prophase I using immunocytochemistry combined with fluorescence light microscopy and SIM. At leptotene in wild type Arabidopsis, the HORMA domain protein ASY1 is detected in chromosome spreads of PMCs as a linear axis-associated signal. This appears to be comprised of a series of alternating regions of higher and lower signal intensity, suggestive of a domainal organization of ASY1 abundance along the chromosome axis [19,45] (Fig 2A and 2C). Analysis of *Atpch2-1* PMCs at leptotene did not reveal any obvious differences, with localization of ASY1 appearing normal (Fig 2B and 2D). Similarly, the cohesin complex protein SYN1 [46,47] (S7A and S7B Fig) and the chromosome axis protein ASY3 [19] (S7C and S7D Fig) appeared unaffected in *Atpch2-1*, with both forming a linear axis-associated signal from leptotene through to mid-prophase I. That SYN1 localization was normal supported the cytological observation that there was no evidence of a sister chromatid cohesion defect. Consistent with these observations, comparison of the mean total axis length per PMC at leptotene was not significantly different to wild type (*Atpch2-1*: 229 μm versus wt: 220 μm, n = 10, P = 0.53).

Previous immunolocalization studies show that the Arabidopsis SC TF protein ZYP1 begins to polymerize between the aligned homologous chromosomes from multiple sites of synapsis initiation at the onset of zygotene. Polymerization continues throughout zygotene until completion of SC formation at pachytene [20]. Dual-localization of ZYP1 and ASY1 in wild type Arabidopsis revealed that SC formation is accompanied by a reduction in the intensity of ASY1 signal which appeared less continuous and appeared to be associated with the chromatin loops rather than the axis along synapsed regions (Fig 2E–2J: compare synapsed segment with unsynapsed region in 2G and 2J; S8A–S8C Fig). Quantification of the relative intensity of the ASY1 signal (S9 Fig) indicated a significant reduction of 67.0% (n = 23; P = <0.001) on the synapsed region compared to the unsynapsed axes (S9C and S9D Fig; S1 Table). Analysis of *Atpch2-1* PMCs at mid/late-prophase I suggested that unlike wild type, the ASY1 signal intensity along the synapsed compared to unsynapsed regions remained unchanged (n = 22; P = 0.25) (S9E and S9F Fig; S1 Table). However, the differentiation of the ASY1 signal into putative domains of high and low intensity appeared enhanced in the mutant, possibly a consequence of the delayed synapsis and increased axis compaction relative to leptotene (S9F Fig). SC polymerization was compromised in the mutant (Fig 2M–2P). Stretches of ZYP1 were detected but varied in number and length from cell to cell. On average the SC length at late prophase I in *Atpch2-1* was 32% that of wild type (57 μM, n = 16 versus 179 μM, n = 8), although this ranged from 13% to 57%.

### PCH2 distribution during prophase I

**Fig 3:**
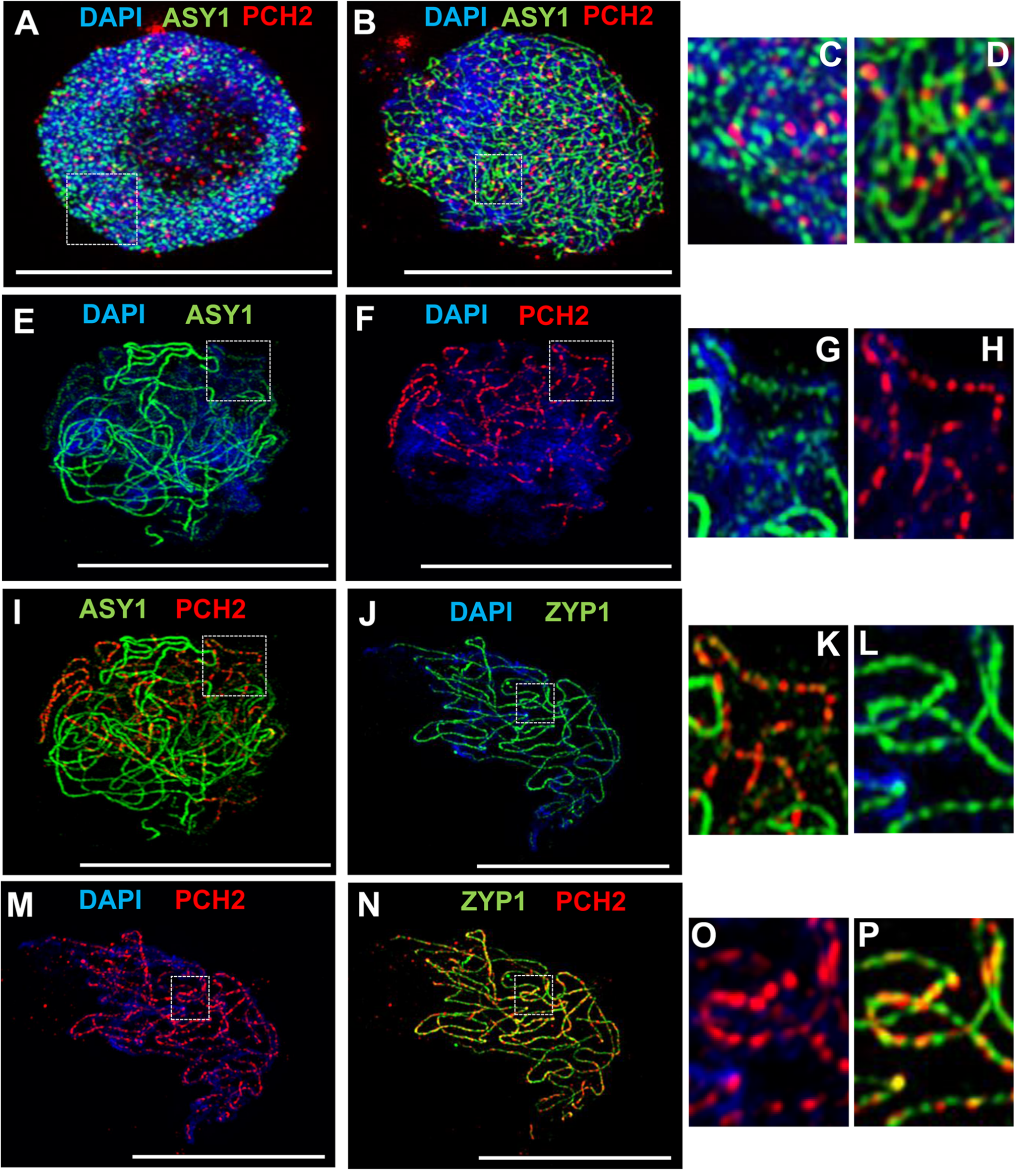
Immunolocalization of PCH2 in wild type Arabidopsis. **(A-D)** Dual localization of ASY1 (green) and PCH2 (red) on chromosome spread preparations from wild type PMCs at G2 **(A)** and leptotene **(B)**. Panels (**C)** and (**D)** show magnified sections of **(A)** and **(B)** respectively. **(E-I,K)** Immunolocalization of ASY1 (green) and PCH2 (red) and merge **(I,K)** in wild type at mid-prophase I. Panels **G, H** and **K** show magnified sections of axes from images **E, F** and **I** respectively. **(J,L-P)** Immunolocalization of ZYP1 (green) and PCH2 (red) and merge **(N,P)** in a wild type nucleus at mid-prophase I. Panels **L, O** and **P** represent magnified sections of axes from **J, M** and **N** respectively. DNA is stained with DAPI. Bar = 10 cm.

**Fig 4:**
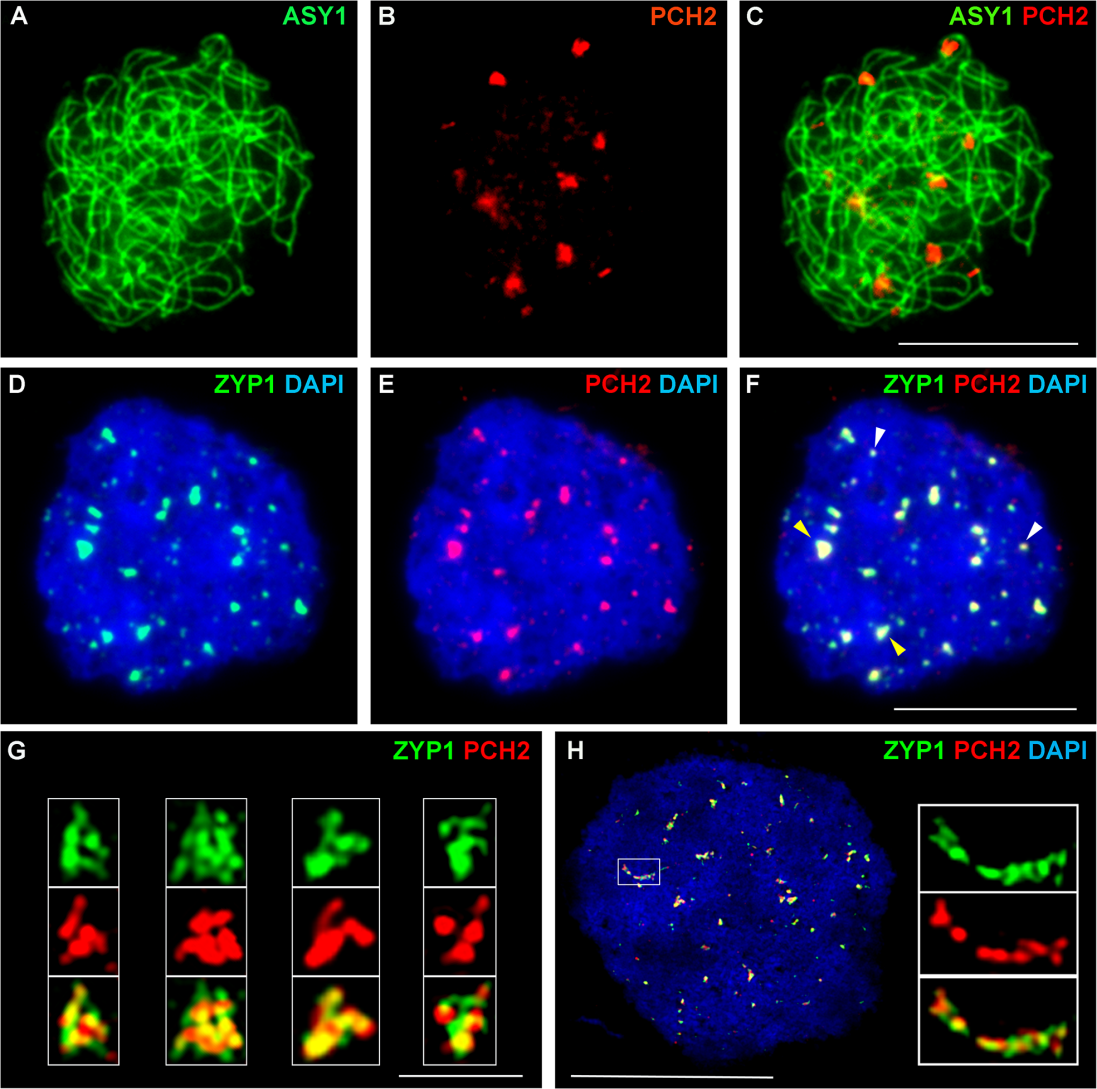
Immunolocalization of PCH2 in *B*. *oleracea* at the leptotene/zygotene transition. **(A-C)** Dual localization of ASY1 (green) and PCH2 (red) on chromosome spread preparations from *B*. *oleracea* PMCs at the leptotene/zygotene transition. **(D-F)** Dual localization of ZYP1 (green) and PCH2 (red) at SC nucleation sites. Yellow arrows indicate examples of large ‘arrowhead’ shaped foci and white arrows indicate the smaller SC nucleation sites. **(G)** SIM images of arrowhead SC nucleation sites stained with ZYP1 (green) and PCH2 (red). **(H)** SIM image of dual localization of ZYP1 (green) and PCH2 (red) on a nascent stretch of SC. DNA is stained with DAPI (blue). Bar = 1 μm in **(G)** and 10 μm in all other images.

To gain further insight into the relationship between PCH2 and the components of the chromosome axes, we conducted immunolocalization studies using an anti-PCH2 antibody on chromosome spreads of wild type PMCs from Arabidopsis and *B*. *oleracea* (Figs 3 and 4). Analysis of Arabidopsis using SIM revealed numerous chromatin-associated PCH2 foci (mean 165; n = 10) in G2 coinciding with the appearance of foci and short stretches of ASY1 (Fig 3A). Most PCH2 foci remained distinct from the ASY1 signal (Fig 3A, inset 3C). As the chromosome axis formed in leptotene, the ASY1 signal became more linear. At this stage the proteins appeared associated, with 51.2% (n = 12) of the PCH2 foci overlapping the ASY1 signal to some extent (Fig 3B, inset 3D), possibly a consequence of the chromosome reorganization that occurs at leptotene. As the SC formed during zygotene PCH2 distribution changed. ASY1 associated foci were no longer apparent. Instead PCH2 now tracked the depleted ASY1 signal along the synapsed region, forming a linear array of foci that tended to coalesce (Fig 3E and 3I). Dual-immunolocalization of PCH2 and ZYP1 confirmed that the PCH2 signal localized to the regions where SC nucleates and was present as foci along the SC during zygotene through pachytene (Fig 3J–3P and S10A–S10C Fig). Analysis in *Atasy1* and *Atasy3* mutants where SC formation is severely compromised, such that only short stretches or accumulations of ZYP1 are formed [19,23], also revealed colocalization of the PCH2 and ZYP1 signals (S10D–S10I Fig). No PCH2 signal was detected in any of the three *Atpch2* mutant lines (S11 Fig).

Immunolocalization in *B*. *oleracea* PMCs revealed that similar to Arabidopsis, numerous PCH2 foci were detected in late G2/early leptotene (S12A and S12B Fig). At late leptotene/early zygotene PCH2 formed fewer, large foci (mean number per nucleus = 14.2; range = 10–20; n = 18) (Fig 4A–4C). Dual localization of ZYP1 and PCH2 at this stage indicated that these foci correspond to sites of SC nucleation at the leptotene/zygotene transition (Fig 4D–4F) and SIM analysis revealed that the ZYP1 signal at the nucleation site often appeared to form a ‘arrowhead-like’ shape to which PCH2 co-localized (Fig 4G). From the SIM images the arrowhead-like foci were estimated to have a mean length of 602 nm (range = 560–640 nm; n = 40) and a mean maximum width of 419 nm (range 400–480 nm; n = 40) and seemed quite consistent in number (mean 15 per nucleus; range = 12–19; n = 5). In a larger sample, analysed using fluorescence microscopy, a mean of 12.2 foci per nucleus was observed (n = 50). Although the range (5–22) was greater than in the SIM sample, most nuclei (76.0%) contained 10 or more foci. The slight variation in the number of PCH2 foci observed in these experiments probably reflected the dynamics of the process and the increased resolution afforded by SIM relative to fluorescence microscopy. In addition to the large foci, slightly more numerous smaller ZYP1 foci were also observed at early zygotene (mean number per nucleus = 17.0; n = 50). These also co-localized with PCH2 (Fig 4D–4F). As the SC began to extend, SIM revealed extensive overlap between ZYP1 and PCH2 signals each appearing to be comprised of multiple smaller foci (Fig 4H). At zygotene, the ASY1 signal appeared to be reduced along synapsed regions of the chromosomes (S12C Fig) (64.3% reduction relative to unsynapsed axes; n = 14), which were decorated with numerous small PCH2 foci. At pachytene, PCH2 foci were still detected along the entire length of the ZYP1-stained SC as well as in the surrounding chromatin (S12D Fig).

### Prophase I progression is delayed in *Atpch2-1*

In budding yeast deletion of *PCH2* results in an accumulation of nuclei in pachytene and a delay in progression through meiosis I [27]. We therefore investigated if the protein has a role in prophase I progression in Arabidopsis. 5-ethynyl-2’-deoxyuridine (EdU) was used to pulse-label *Atpch2-1* PMCs during meiotic S-phase [48]. Progression through meiosis was then monitored (S13A Fig). In wild type and *Atpch2-1*, EdU labelled leptotene nuclei were detected 10h post S-phase. By 25h all labelled wild type PMCs were at zygotene or pachytene and at zygotene in *Atpch2-1*. At 32h the wild type PMCs had exited pachytene and were at diplotene/diakinesis and by 36h were at the dyad stage, whereas *Atpch2-1* PMCs were still at zygotene suggesting a delay of 5-8h (S13A and S13B Fig).

### Fluorescent tetrad analysis supports a CO defect in *Atpch2-1*

**Fig 5:**
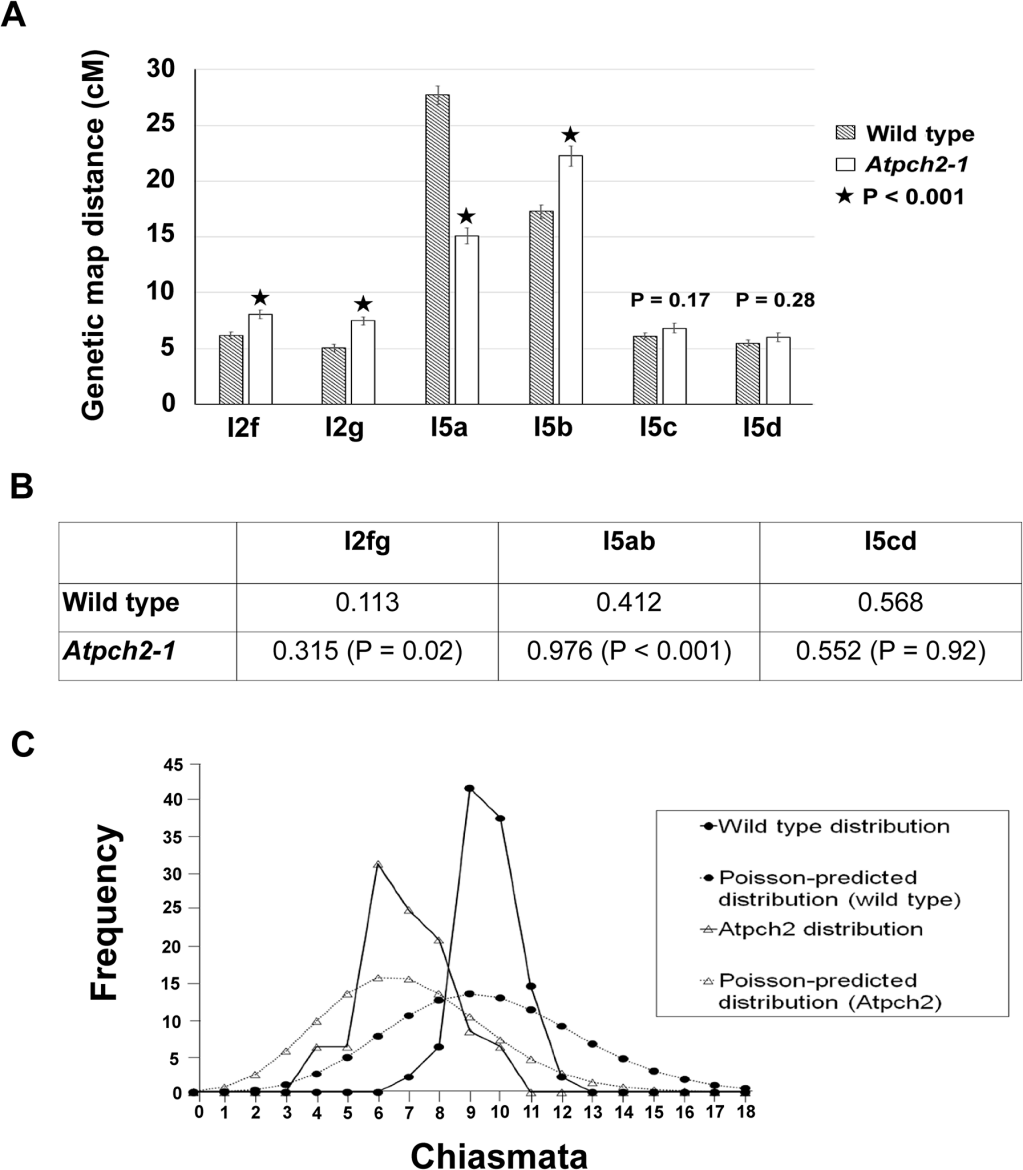
Recombination frequency and chiasma distribution in *Atpch2-1*. **(A)** Genetic map distance of four distinct intervals on chromosome 5 and two intervals on chromosome 2 in wild type Arabidopsis and *Atpch2-1* mutant. Black stars represent a statistical difference in the genetic map distance between wild type and mutant. P value is indicated on the graph when the genetic map distance of an interval is not statistically different between wild type and mutant. Error bars represent the standard error of the mean. **(B)** Three pairs of adjacent intervals l2fg, I5ab and I5cd were used to estimate genetic CO interference for wild type and *Atpch2-1* mutant. CO interference ratio for each pair of intervals is indicated in the table and P value is shown in parentheses. **(C)** Observed (solid line) and Poisson-predicted (dotted line) distributions of chiasma numbers per cell for wild type (black circle) and *Atpch2-1* mutant (white triangle).

The cytological analysis (see earlier) suggested a defect in CO formation in *Atpch2* mutants. To further examine the recombination phenotype of *Atpch2-1* we used the fluorescent-tagged-line (FTL) system [49,50] which relies on the segregation of three genetically linked transgenic markers, each encoding a distinct pollen-specific fluorescent protein expressed post-meiotically. The FTLs are in a *qrt1-2* mutant background which prevents the separation of the gametes and facilitates the visualisation of the meiotic recombination events that have occurred between the transgenic markers in the tetrad pollen [51,52]. Three pairs of adjacent genetic intervals, one on each arm of chromosome 5 and another on chromosome 2 were examined (S14 Fig). This revealed that the genetic map distance determined using the Perkins mapping equation [53] in the adjacent intervals I5c and I5d was not significantly affected by the *Atpch2-1* mutation (I5c wild type 6.1 cM v *Atpch2-1* 6.8 cM; P = 0.17; I5d wild type 5.5 cM v *Atpch2-1* 6.0 cM; P = 0.28) (Fig 5A). However, interval I5a showed a significant decrease in recombination frequency in the presence of *Atpch2-1* (15.1 cM) compared to wild type (27.7 cM; P < 0.001), whereas the map distance in interval I5b exhibited a significant increase from 17.3 cM in wild type to 22.3 cM in the mutant (P < 0.001) (Fig 5A). A significant increase in map distance was observed in intervals l2f and l2g in the presence of the *Atpch2-1* mutation (l2f wild type 6.1 cM / *Atpch2-1* 8.0 cM P < 0.001; l2g wild type 5.1 cM / *Atpch2-1* 7.1 cM P < 0.001) (Fig 5A).

We used the FTL data to obtain a genetic estimate for CO interference in adjacent intervals by calculating the Interference Ratio (IR). This method, developed by Malkova et al. [54], uses the ratio of the genetic map distance in an interval with and without the presence of a CO in an adjacent interval to provide an estimate of the strength of CO interference. When COs in adjacent intervals are entirely independent of each other the IR is 1, indicating no interference. Values less than 1 indicate increasing levels of (positive) interference with a value of 0 indicating complete interference. IR ratios greater than 1 are indicative of negative interference. The CO interference ratio was 0.412 for I5ab in wild type. In *Atpch2-1*, the genetic map distance of I5a was similar with and without the presence of a CO in the interval I5b (14.8 cM with a CO in interval I5b vs 15.2 cM without a CO in interval I5b). The CO interference ratio is 0.976 and is statistically higher than wild type (Z-score = 5.40; P < 0.001) (Fig 5B). This suggests that CO interference is reduced in the interval I5ab in *Atpch2-1*. In contrast, the CO interference ratio of I5cd is similar in wild type (0.568) and in *Atpch2-1* (0.552; Z-score = 0.01; P = 0.92) (Fig 5B). The interference ratio for interval l2fg is also increased in the *Atpch2-1* mutant. In wild type the ratio is 0.113 whereas in *Atpch2-1* it is 0.315 (P = 0.021).

We also used the FTL data to estimate the coefficient of coincidence (CoC) for the three pairs of intervals. The CoC is calculated by dividing the observed frequency of double COs in two adjacent intervals by the expected frequency assuming no interference [55]. When interference is absent the CoC is 1 and where it is complete the CoC is 0. The overall result was similar to that obtained for the IR (S2 Table). For l5a/b interference appeared reduced (CoC wild type = 0.46 v CoC *Atpch2-1* = 0.99), for l5c/d it was unchanged (CoC wild type = 0.60 v CoC *Atpch2-1* = 0.60) and for l2fg there was an apparent decrease (CoC wild type = 0.13 v CoC *Atpch2-1* = 0.37).

### Numerical distribution of chiasmata in *Atpch2-1*

In the absence of CO control the numerical distribution of chiasmata between cells is predicted to fit a Poisson distribution [56]. This expectation is borne out in ZMM mutants such as *Atmsh4* and *Atmer3*, whereas in wild type the distribution is non-Poissonian. [43,57,58]. We analysed the chiasma distribution in the sample of *Atpch2-1* cells described above. The number of chiasmata per nucleus ranged between 4 and 10 in *Atpch2-1* and between 7 and 12 in wild type. Further inspection revealed that the proportion of *Atpch2-1* PMCs with a chiasma frequency close to the mean of 6.9 was over-represented in the sample analysed, with 74% having between 6 and 8 chiasmata per cell (vs 42.8% if the numerical distribution of chiasmata was random) (Fig 5C). Over-distribution of chiasma around the mean is also a feature of wild type [43]. The chiasma distribution in *Atpch2-1* differed significantly from a Poisson distribution (X_(11)_^2^ = 45.2; P < 0.001). This was also confirmed in *Atpch2-2* (X_(11)_^2^ = 37.2; P < 0.001) and *Atpch2-3* (X_(11)_^2^ = 40.00; P < 0.001).

### Later stages of recombination are aberrant in *Atpch2-1*

**Fig 6:**
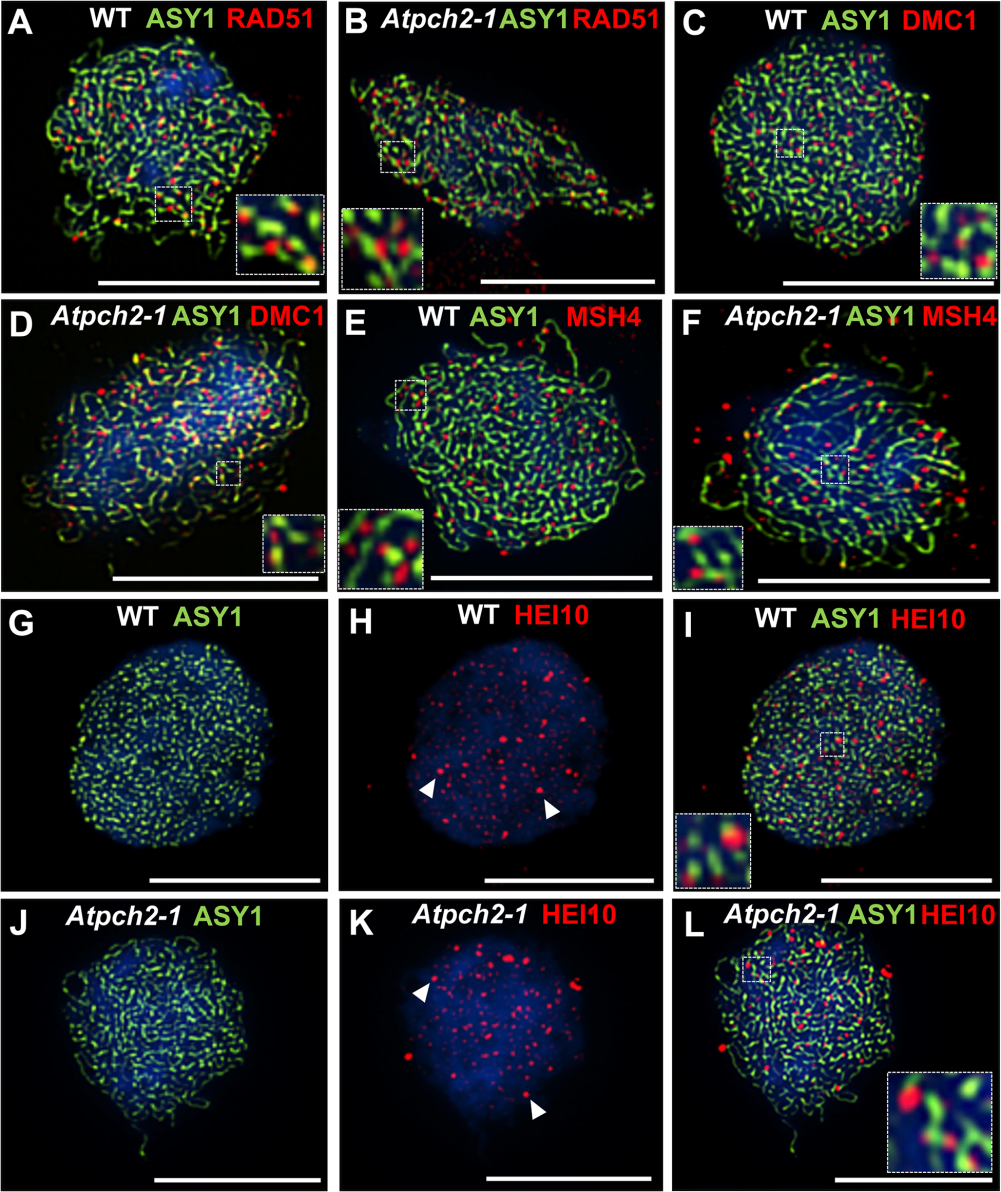
Dual localization of ASY1 and recombination pathway proteins in wild type and *Atpch2-1* meiotic nuclei at early prophase I. Dual localization of ASY1 (green) and RAD51 (red) on wild type **(A)** and *Atpch2-1* **(B)** PMCs at mid-leptotene; ASY1 (green) and DMC1 (red) on wild type **(C)** and *Atpch2-1* **(D)** PMCs at mid-leptotene; ASY1 (green) and MSH4 (red) on wild type **(E)** and *Atpch2-1* **(F)** PMCs at leptotene/zygotene transition; ASY1 (green) and HEI10 (red) on wild type **(G-I)** and *Atpch2-1* **(J-L)** PMCs at late-leptotene. DNA is stained with DAPI (blue). Bar = 10 μm.

We investigated the basis for the reduction in chiasmata in *Atpch2-1* using immunolocalization of recombination pathway proteins on prophase I chromosome spreads from *Atpch2-1* PMCs. Immunolocalization of the strand-exchange proteins RAD51 and DMC1 which are recruited to DSBs at leptotene was used to monitor early recombination and immunolocalization of the ZMM protein AtMSH4 was used to detect later recombination progress [43,59,60]. There were no significant differences between wild type (Fig 6A, 6C and 6E) and *Atpch2-1* (Fig 6B, 6D and 6F) PMCs. At mid-leptotene the mean number of RAD51 foci in *Atpch2-1* was 144 versus 146 in wild type (n = 12; P = 0.37) (Fig 6A and 6B). For DMC1 the corresponding values were 167 versus 173 (n = 12, P = 0.56) (Fig 6C and 6D). In PMCs at the leptotene/zygotene transition the mean number of MSH4 foci was 150 in *Atpch2-1* versus 152 in wild type (n = 12; P = 0.63) (Fig 6E and 6F).

**Fig 7:**
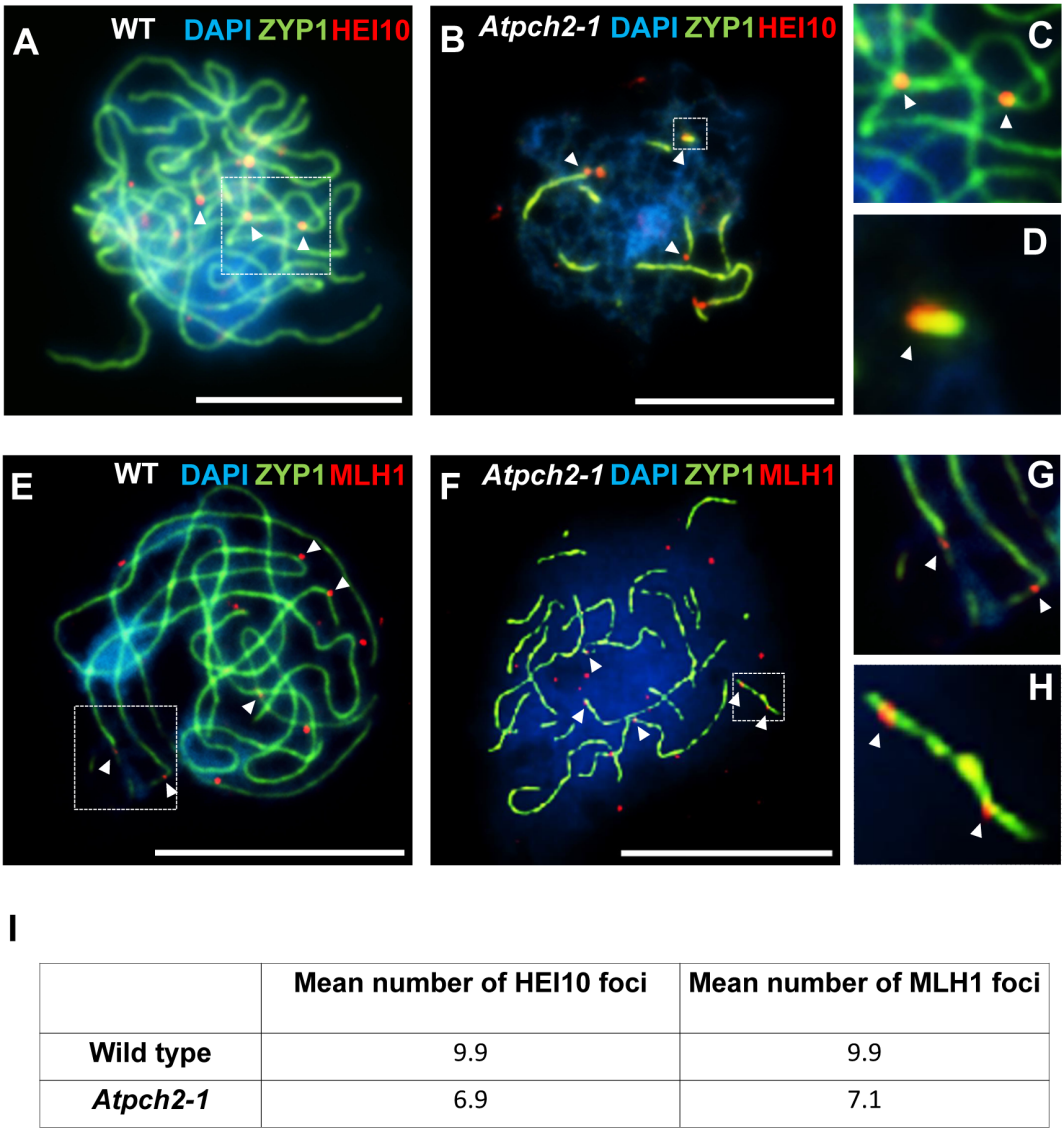
Dual localization of ZYP1 and recombination pathway proteins in wild type Arabidopsis and *Atpch2-1* meiotic nuclei at mid/late prophase. **(A-D)** Dual localization of ZYP1 (green) and HEI10 (red) on wild type **(A,C)** and *Atpch2-1* **(B,D)** PMCs. Panels **C** and **D** show magnified sections of SC from **A** and **B** respectively. **(E-H)** Dual localization of ZYP1 (green) and MLH1 (red) on wild-type **(E,G)** and *Atpch2-1* **(F,H)** PMCs. Panels **G** and **H** show magnified sections of SC from **E** and **F** respectively. DNA is stained with DAPI (blue). Bar = 10 μm. **(I)** Table showing the mean number of HEI10 and MLH1 foci in wild type and *Atpch2-1*.

HEI10 (Human enhancer of invasion-10) is a member of the Zip3/Hei10 family of proteins which are thought to possess SUMO/ubiquitin E3 ligase activity [61]. Studies reveal that Zip3/Hei10 marks the sites of future type I COs [61,62]. In *Sordaria macrospora* Hei10 foci that mark COs are ~300 nm in size and emerge from a much larger population of small axis-associated foci during early/mid-prophase I [63]. Dual localisation of ASY1 and HEI10 on chromosome spreads of Arabidopsis wild type and *Atpch2-1* PMCs at late leptotene showed that in both cases HEI10 formed a very similar large number of foci (166 versus 165 respectively, n = 10; P = 0.81) along the chromosome axes (Fig 6G–6L). As prophase I progressed the foci decreased in number and disappeared by pachytene. Most foci were small (~175 nm) but in addition, a number of larger (>250 nm) HEI10 foci were observed in both sets of PMCs. In wild type at the leptotene/zygotene transition we observed 7 to 15 large HEI10 foci (mean 10.6, n = 33). This remained constant through late pachytene (mean 9.9 range 9–12, n = 14) (Fig 7A, 7C and 7I). During early prophase I the number and distribution of large HEI10 foci in *Atpch2-1* PMCs was not significantly different to wild type (mean 10.6 versus 10.2; P = 0.20; n = 21). However, at mid/late prophase the mean number of large HEI10 foci was significantly reduced to 6.9 (n = 27) compared to wild type nuclei (P < 0.001) (Fig 7B, 7D and 7I). HEI10 foci were mostly found as singletons on the stretches of SC in *Atpch2-1* (83.4% n = 185) (Fig 7B and 7D), with two or three HEI10 foci observed in 14.6% and 2.0% of cases respectively.

Dual localization of HEI10 and ZYP1 in *B*. *oleracea* PMCs at the leptotene/zygotene transition revealed that most of the large ZYP1 foci at SC nucleation sites that had been shown to co-localize with PCH2 at this stage (see earlier), also co-localized with HEI10 (86.0% foci; n = 30 nuclei) (S15 Fig).

To confirm that the reduction in large HEI10 foci in *Atpch2-1* reflects a reduced number of mature CO intermediates we analysed the distribution of the late recombination protein MLH1 which marks the sites of Type I COs/chiasmata [64]. Dual immunolocalization of MLH1 and ZYP1 (N-terminus Ab, see Materials and Methods) on chromosome spreads of wild type PMCs, showed the number of MLH1 foci per nucleus varied between 9 and 11 with a mean count of 9.9 (n = 12) at pachytene (Fig 7E, 7G and 7I). In *Atpch2-1* the mean number of MLH1 foci per nucleus was 7.1 (n = 12), a significant reduction compared to wild type (P < 0.002) (Fig 7F, 7H and 7I). We noted that in both cases the MLH1 foci were often adjacent to the ZYP1 signal rather than directly over the SC central region. Similar to the distribution of HEI10 foci, MLH1 foci were mostly observed as singletons on stretches of SC in *Atpch2-1* PMCs (61.2% n = 85) with two or three foci occurring in 28.2% and 10.6% cases respectively (Fig 7F and 7H).

## Discussion

### Chromosome axis remodeling at the leptotene/zygotene transition is defective in the absence of PCH2

Formation of the chromosome axis in early prophase I appears unaffected by loss of PCH2 based on immunolocalization of the axis proteins and axis length measurements. This differs from rice where the PCH2 ortholog, CRC1, is required for recruitment of the ASY1 ortholog PAIR2 onto the chromosome axes at leptotene [38]. This difference between the two plant species is perhaps surprising but it is not the first example where the phenotype of a rice meiotic mutant is different to that in other plants. For instance, loss of *ZYP1* in Arabidopsis and barley results in a reduction of CO formation whereas mutation of the corresponding rice gene, *ZEP1*, leads to increased COs [20,65,66].

At mid-prophase I in Arabidopsis and *B*. *oleracea*, PCH2 forms foci along the SC which correlate with regions of ASY1 signal depletion on the axes. The overall distribution of PCH2, together with the fact that ASY1 signal intensity is not reduced along the synapsed axes in *Atpch2* mutants, suggests that PCH2 participates in the depletion of ASY1 from the axis at the leptotene/zygotene transition. This could be a direct effect since biochemical studies in budding yeast show that Pch2 can bind to Hop1 *in vitro* and binding is strongly enhanced if its ATP hydrolysis activity is blocked [67]. In addition, Pch2 was shown to displace Hop1 from double-stranded DNA. Direct interaction *in vivo* has not been established as it is argued that this would be transient in the presence of ATP [67]. Based on the number of peptides recovered, PCH2 is found as an abundant component of a complex that is co-precipitated with ASY1 from Brassica PMCs. Although this could reflect a direct interaction, PCH2 may be co-precipitated as part of a larger chromosome axis-protein complex. Thus, an alternative possibility is that the reduction in the ASY1 signal is an indirect consequence of PCH2-dependent reorganization of the chromosome axis at the onset of zygotene.

### PCH2 is important for synapsis but does not appear to be an integral SC component

In rice, loss of the PCH2 ortholog, CRC1 leads to a failure to form SC. This is unsurprising given that DSBs are not formed in a *crc1* mutant [38]. Nevertheless, studies indicate that CRC1 localizes to the central region of the SC at pachytene and interacts with the SC transverse filament protein ZEP1 in a yeast two-hybrid assay, suggesting it is a component of the SC [38]. Analysis of the *Atpch2* mutants indicates that PCH2 plays a critical role in formation of the SC, since loss of the protein results in a substantial defect in polymerization of the SC transverse filament protein ZYP1. An average reduction in SC length of 68% was observed but this was quite variable ranging from 43% to 87%. Similar to rice, co-localization between PCH2 and ZYP1 is also observed from the beginning of zygotene through pachytene. In *Atasy1* and *Atasy3* mutants, where SC polymerization is compromised, PCH2 is associated with the residual ZYP1 signal. Association of ZYP1 and PCH2 is also supported by SIM analysis of the *B*. *oleracea* SC as it begins to extend, although this suggests that they are not forming a homogeneous complex. This could reflect that any interaction between the proteins is transient. Since ASY1 appears to be the target for PCH2, it is conceivable that ZYP1 or another component of the SC central region acts to couple/guide the PCH2/ASY1 interaction. A precedent for this is seen in the bacterial P1 plasmid partitioning system in which the ParA ATPase is functionally coupled by the ParB protein to move its plasmid DNA cargo via a diffusion-rachet mechanism [68]. Furthermore the interaction between the *C*. *elegans* PCH2 ortholog, PCH-2, and the HORMAD spindle checkpoint protein Mad2, has been shown to involve an adaptor protein, p31 [69].

The SC nucleations seen in *B*. *oleracea* were consistent in size and their arrowhead-like shape is likely a consequence of the convergence of the homolog axes at the synaptic site. It is noteworthy that in most nuclei examined the number of large foci is broadly similar to the chiasma frequency (13–15) in *B*. *oleracea* [70,71]. Moreover most of the arrowhead-like ZYP1 foci (86.0%) co-localized with HEI10, a further indication that they occur at designated CO sites. Nuclei with fewer large foci may have been at a slightly earlier stage and reflect the dynamic nature of the initial appearance of foci. The smaller, slightly more numerous ZYP1 foci that were also present at early zygotene are likely to be additional synapsis initiation sites. The apparent existence of two classes of SC nucleation structures is reminiscent of observations in *S*. *macrospora* [72]. These have revealed distinct types of designations, one defining SC nucleation sites that correspond to CO designated recombination events and another that defines a similar number of sites where SC nucleation alone occurs. Importantly, the distribution of both classes exhibit interference and fits the prediction of the ‘beam-film’ model [11,12]. This posits that mechanical stress arises within a chromatin-axis meshwork as a result of global chromatin expansion during leptotene. Subsequent bi-directional relief of this stress results in a set of CO designations (and SC nucleations) that are spatially separated along the chromosomes. Further studies will be required to establish if the observed SC nucleations in *B*. *oleracea* also reflect a corresponding underlying interference-dependent distribution.

In other species loss of PCH2 orthologs leads to a variety of different effects on synapsis. In mouse, mutation of the Pch2 ortholog TRIP13 also results in a synaptic defect, albeit less severe than in Arabidopsis, with the unsynapsed regions accounting for just under 30% of the total axis length [34]. Loss of Pch2 in budding yeast does not appear to affect SC formation [27]. However, budding yeast forms high levels of COs and each designated CO site is thought to nucleate SC formation [73]. Hence, loss of Pch2 may not impact on SC installation to the degree observed in Arabidopsis where the relative CO rates are far lower. PCH2 also impacts on SC formation in *C*. *elegans* but in this case SC formation occurs more quickly than in wild type [37]. Interestingly, this defect was suppressed at lower temperatures. Thus loss of Pch2 has differing effects on the extent of SC polymerization in different species but in each case is associated with a recombination defect. Together, these observations suggest that PCH2 is not an integral structural component of the SC and more likely, regulates the coordination of synapsis with the controlled formation of COs.

### Early recombination pathway events appear normal and DSBs are repaired in *Atpch2-1*

The controlled formation of COs via homologous recombination is an essential feature of meiosis. Studies of PCH2 in several species have linked loss of the protein to a variety of recombination defects. In the most severe case, loss of the rice PCH2 ortholog, CRC1, is reported to result in a failure to form DSBs [38]. In budding yeast, a minor role for Pch2 in DSB formation has been reported [74]. It is also involved in processing of early occurring, low abundance DSBs and loss of the protein leads to a coordinate delay in the repair of DSBs to form both CO and NCO products [27,28].

In mouse, studies suggest that a severe reduction in TRIP13 expression does not compromise DSB formation but loading of RAD51 onto the resected DSBs is reduced [34]. In Arabidopsis, immunolocalization of RAD51 and DMC1 in *Atpch2-1* PMCs indicated that early stages in recombination occur normally. As there is no evidence of chromosome fragmentation, it seems DSBs are also repaired, albeit with a reduction in CO formation, but progression through prophase I is delayed by 5-8h. This is reminiscent of that seen in some meiotic mutants and is indicative of an underlying defect in the recombination pathway [20,43,64]. Since a significant reduction in CO frequency was observed in an *Atpch2/Atmsh5* double mutant relative to an *Atmsh5* mutant it appears that loss of PCH2 impacts on the formation of both Class I and Class II COs.

### CO interference is likely established in *Atpch2-1* but maturation of designated COs appears defective

Studies in different species have reported a CO interference defect associated with mutation of *Pch2/TRIP13*. Genetic analysis in budding yeast using intervals across a range of chromosomes of different sizes has revealed an increased frequency of closely spaced double-CO events in the absence of Pch2 [28,31]. In a *Trip13* hypomorphic mutant mouse, despite an overall reduction in MLH1 foci at pachytene, a small, yet significant, reduction in the mean inter-focus distance between pairs of foci was observed. This implies that although the COs remain subject to interference, there has been some weakening in its effect, although a subtle change in the positioning of the DSB complexes cannot be excluded [34]. Despite these observations recent evidence from budding yeast has found that inter-focus distance of Zip3 foci that mark future COs is not affected by loss of Pch2, indicating CO interference is normal [32]. Why the discrepancy? Zip3 foci are the earliest known marker of CO designation, appearing in late leptotene. However, maturation of designated intermediates to form COs is dependent on additional later events during the remainder of prophase I [5]. Other analyses of *pch2* mutants have used genetic markers or MLH1 foci, which mark mature CO sites. Hence it is conceivable that while loss of Pch2/Trip13 affects the final CO patterning, CO designation occurs and hence interference is initially established. Analysis of *Atpch2-1* is consistent with this possibility. The localization of HEI10 foci in wild type and *Atpch2-1* at early prophase I was identical. Numerous small axis-associated foci were observed together with around 10 large (~250 nm) foci. At present, it is not technically possible to measure inter-focus distance at early prophase I in Arabidopsis, nevertheless inspection of the nuclei reveals these large HEI10 foci are usually spatially well separated. These are still observed at mid/late prophase I when a similar number of MLH1 foci, which mark interference sensitive CO sites, are also observed. By analogy with budding yeast and *S*. *macrospora* where the appearance of Zip3/Hei10 foci are indicative of CO designation in early prophase I, it seems likely this is also the case in Arabidopsis as the number of HEI10 foci at the leptotene/zygotene transition appeared normal in the absence of PCH2. However, the maturation of the CO designated intermediates is compromised by the defect in remodelling of the chromosomes axes in *Atpch2-1*, leading to a deficit in COs. Overall, our data imply that in Arabidopsis, as in budding yeast and *S*. *macrospora*, CO designation and interference arise, and are complete, during zygotene.

We noted that in both wild type and *Atpch2-1* some MLH1 foci appeared adjacent to the ZYP1 signal rather than directly over it. This has not been previously recorded in Arabidopsis. It may be a consequence of the spreading procedure but it is worth noting that in this study, the anti-ZYP1 antibody was raised to the N-terminus of the protein which is predicted to mark the central region of the SC. This could suggest the MLH1 containing complexes are not in direct contact with the SC central region. However the basis and significance of this remains unclear.

Other features of the CO distribution in the mutants indicate that CO interference is established. A predicted outcome of CO interference is that the numerical distribution of interference-sensitive COs between nuclei does not fit a Poisson distribution, whereas the converse applies for non-interfering COs [56]. The distribution of COs in *Atpch2-1* does not fit a Poisson distribution, suggesting that COs remain subject to spatial patterning and do not arise by the random maturation of a proportion of the recombination initiations into COs. Also, there was a strong tendency for any HEI10 or MLH1 foci that were found associated with stretches of SC in *Atpch2-1* to occur as single foci.

Although the data indicate that CO designation occurs normally, it seems that precursor maturation to form CO products is perturbed in *Atpch2*. This is manifested in several ways. Most obviously, the mean chiasma frequency in *Atpch2* mutants is ~7, a reduction of around 30% relative to wild type. This is accompanied by the presence of univalents at metaphase I at a frequency of ~10%. A global reduction in CO formation has also been reported in TRIP13/Pch2-deficient mice and PCH2-deficient *C*. *elegans* [34,37]. In budding yeast, an increase in COs has been reported for some genetic intervals whereas in others wild type levels were recorded [31]. Also, the distribution of MLH1 foci in the mouse *Trip13* mutants suggests there are chromosomal regions which show an increase in CO frequency [34]. This could suggest variation between different species but it is worth noting that despite the global reduction in COs in the absence of PCH2 an increase in recombination frequency was observed in 3 out of 6 intervals (l2f, I2g and 15b) in the Arabidopsis FTL lines used in this study. However, this comes with the caveat that this approach scores only viable tetrads which could influence the analysis. Estimation of genetic CO interference using the FTL lines suggested its effect may be diminished in at least some chromosomal regions, since a reduction in strength was detected over regions of chromosome 2 and chromosome 5. This apparent contradiction with the cytological evidence can perhaps be reconciled by data from a study of CO patterning in *S*. *macrospora* applying the beam-film model to experimental data. This showed that under some circumstances a normal interference signal is established and remains, yet CO interference as measured using CoC as a metric appears to be reduced [7,72].

An altered pattern of COs combined with a synaptic defect and a reduction in genetic interference has been reported in a kinesin mutant, *Atpss1*, and *Ataxr1*, a mutant in the E1 enzyme Arabidopsis neddylation complex [55,75]. Both mutants are strongly defective in synapsis with univalents observed at metaphase I. In each case HEI10 and MLH1 foci are observed in late prophase I in approximately wild type numbers but, in contrast to *Atpch2-1*, often clustered along the limited stretches of SC that have formed. An effect on the distribution of MLH1 foci has also been reported in *as1*, an asynaptic mutant of tomato [76]. The genetic basis of the *as1* mutation is unknown but it is associated with changes in compaction of the chromosome axes. Relative to wild type, the average SC length in *as1* was reduced by 81% with MLH1 inter-focus distance decreased by 71%. However the median number of MLH1 foci was unchanged, although the range was more variable. It is hypothesized that the tendency of these plant mutants to maintain CO numbers may reflect a homeostatic mechanism [76]. This is not so obvious in *Atpch2-1* but it was notable that the mean reduction in CO frequency (~30%) was not-coordinate with that in SC length (~68%).

This study demonstrates that in the absence of PCH2, remodelling of the chromosome axis at zygotene and the normal patterned maturation of CO designated intermediates in Arabidopsis are aberrant. This further emphasises the functional inter-relationship between the chromosome axis and the controlled formation of COs.

## Materials and Methods

### Plant material and nucleic acid extraction

*A*. *thaliana* ecotype Columbia (0) was used for wild type analysis. T-DNA insertion lines *Atpch2-1*: SAIL_1187_C06, *Atpch2-2*: SALK_031449 and *Atpch2-3*: SALK_130138 were obtained from NASC for mutant analysis. Plants were grown, material harvested and nucleic acid extractions were performed as previously described by Higgins et al. [43].

### Proteomic analysis of Brassica PMCs

AtPCH2 peptides were identified by mass spectrometry in protein extracts from *Brassica oleracea var*. *alboglabra* A12DHd PMCs following co-immunoprecipitation with affinity purified anti-ASY1 antibody as previously described [40].

### T-DNA insertion site mapping

The T-DNA insertion site of the mutant lines was confirmed as previously described [43]. Details of the primers used are presented in S3 Table.

### RNA extraction and RT-PCR

RNA extraction and RT-PCR was carried out as previously described [43]. Details of the primers are given in S3 Table.

### Nucleic acid sequencing

Nucleotide sequencing was carried out by the Genomics and Proteomics Unit, School of Biosciences, University of Birmingham, UK.

### Antibody production

An anti-PCH2 antibody was raised in rabbit against a 15-residue peptide from the C-terminus of Arabidopsis PCH2 (Abmart Inc., Shanghai, China). Due to the high level of sequence identity between the PCH2 proteins in Arabidopsis and Brassica the antibody was also effective for immunolocalization in Brassica.

### Cytological procedures

Cytological studies were carried out as previously described [43]. The following antibodies were used: anti-AtPCH2 (rat 1/200 dilution), anti-AtASY3 (rabbit, 1/200 dilution) [19], anti-AtASY1 (rabbit/rat, 1/1000 dilution) [45], anti-AtMSH4 (rabbit, 1/500 dilution) [43], anti-AtZYP1 (N-terminus Ab aa residues 1–415; C-terminus Ab aa residues 422–845; rabbit/rat, 1/500 dilution), anti-AtRAD51 (rabbit 1/500 dilution), anti-AtSYN1 (rabbit 1/500 dilution), anti-AtDMC1 (rabbit 1/500 dilution) [20,23], anti-AtMLH1 (rabbit/rat, 1/200 dilution) [64], anti-AtHEI10 (rabbit 1/500 dilution) and anti-γH2AX (ser 139, catalog no. 07–164 Upstate Biotechnology; rabbit, 1/100 dilution). Microscopy was carried out using a Nikon 90i Fluorescence Microscope (Tokyo, Japan). Image capture, image analysis and processing were conducted using NIS-Elements-F software (Nikon, Tokyo, Japan) as previously described [19]. Image deconvolution was carried out using the function “Mexican hat”. This allows better discrimination of the signals. This function performs filtration on the intensity component (or on every selected component—when working with multichannel images) of an image using convolution with 5x5 kernel. Mexican Hat kernel is defined as a combination of Laplacian kernel and Gaussian kernel it marks edges and also reduces noise. SIM was carried out using the OMX facility at the University of Dundee (http://microscopy.lifesci.dundee.ac.uk/omx/).

In Arabidopsis, ASY1 intensity analysis was conducted on chromosome spread preparations stained with anti-ASY1 antibody (rat, 1 in 5000 dilution) and anti-ZYP1 (rabbit, 1 in 500 dilution). 5μl of 6μm, 0.3% relative intensity InSpeck Red microspheres (Life Technologies), were added to slides before coverslips. PMCs and microspheres were imaged using specific exposure times. Randomly selected, non-overlapping sections of axis, ~2–4μm in length, were defined as regions of interest and were analysed for mean signal intensity using Nikon NIS-elements software. Intensities were normalised based on mean intensity of the microspheres. Intensity raw data is shown in grey-scale values. For *B*. *oleracea*, ASY1 intensity was determined in on PMC chromosome spreads at zygotene comparing non-overlapping segments of unsynapsed and synapsed sections of axis ~2–4μm in length.

Chiasma counts were carried out as previously described [42]. Chromosome spread preparations from PMCs at metaphase I were examined by light microscopy after fluorescence *in situ* hybridization (FISH) using 45S and 5S rDNA probes. The use of FISH enabled the identification of individual chromosomes. The overall shape of individual bivalents allowed the number and position of individual chiasmata to be determined and this was also informed by the position of the FISH signals.

The time course of progress through prophase I in wild type and *Atpch2-1* was determined as previously described [48] except that 5-ethynyl-2’-deoxyuridine (EdU) was used to label the PMCs which were analyzed at 5 h intervals from 0–30 h and at 2 h intervals thereafter up to 36 h.

Fluorescent tetrad analysis was carried out as described Berchowitz and Copenhaver [49] using genetic intervals I2f and I2g on chromosome 2 (FTL coordinates for the I2fg interval: FTL#800 18286716 bp DsRed2; FTL#3411 18957093 bp YFP; FTL#3263 19373634 bp AmCyan) and I5a, I5b, I5c, and I5d on chromosome 5 as described [49]. Pollen was scored through eCFP, eYFP and DsRed2 filters using an Olympus BX-61 epifluorescence microscope. The Stahl Lab Online Tools (http://molbio.uoregon.edu/~fstahl/) was used for statistical analyses of the data.

### Statistical procedures

The statistical procedures were carried out as described previously [43]. Chi-squared (Χ^2^) tests were used to determine agreement between the observed chiasma counts and those expected from a Poisson distribution. Numbers of foci in wild type and mutant PMCs were compared using the Wilcoxon signed-rank test. Mean intensities between synapsed and unsynapsed sections of axis/SC were analysed using a 2 tail paired T-test.

## Supporting Information

S1 Fig PCH2 peptide coverage and orthologs. **(A)** Sequence coverage of the predicted protein product of Bra013827 (yellow highlight). **(B)** Cladogram derived from ClustalW2 analysis of AAA+ATPase proteins from *A*. *thaliana*. At4g24710 belongs to a sub-family which also includes Bra013827 and PCH2 homologues from budding yeast (ScPCH2), mouse (MmTRIP13) and rice (OsCRC1). During the course of this work the sequence of two *B*. *oleracea* PCH2 orthologues became available (Liu et al. 2014; Parkin et al. 2014) and are included in the analysis.
(TIF)

S2 Fig Structure and expression of *PCH2*. **(A)** Schematic illustration of Arabidopsis PCH2 protein. *AtPCH2* is predicted to encode a protein of 475 amino acids with a putative AAA-ATPase domain located between amino acids 213 and 358. **(B)** Map of *AtPCH2* locus showing the exon/intron organization. Exons are represented with black boxes. 3’ UTR region is represented with a blue box. Triangles represent the location of T-DNA insertion sites for all three *Atpch2* mutants. Red arrows mark the position of the primers used for detecting the full-length *AtPCH2* transcript by RT-PCR. **(C)** Gene expression analysis of *AtPCH2* using semi-quantitative RT-PCR from wild type and *Atpch2-1*, *Atpch2-2* and *Atpch2-3* bud tissues. The amount of RNA used for each sample was equalized using the housekeeping gene *AtGAPD*. *AtPCH2* was expressed in wild type buds while no full length transcript was detected in *Atpch2* mutants.
(TIF)

S3 Fig Nucleotide sequencing of the T-DNA insertion sites in *Atpch2* mutants. (TIF)

S4 Fig Phenotype of *Atpch2* mutants. **(A)** Vegetative growth is normal but fertility is reduced in *Atpch2* mutants. Bar = 5 cm. **(B)** Silique length is slightly reduced and numerous gaps are observed between the seeds in *Atpch2* mutants. Bar = 1 cm. **(C)** Graph showing the mean seed-set per silique from 50 siliques of wild type Arabidopsis and *Atpch2* mutants. Error bars represent the standard deviation. Black stars represent a mean statistical difference between wild type and mutant.
(TIF)

S5 Fig Reduced fertility and meiotic defects in *Atpch2-2* and *Atpch2-3* mutants. **(A-D)** Meiotic stages in *Atpch2-2* mutant. **(A)** late prophase I; **(B)** metaphase I; **(C)** tetrad; **(D)** metaphase I nucleus labelled with 5S (red) and 45S (green) rDNA probes. **(E-H)** Meiotic stages in *Atpch2-3* mutant. **(E)** late prophase I; **(F)** metaphase I; **(G)** tetrad; **(H)** metaphase I nucleus labelled with 5S (red) and 45S (green) rDNA probes. Bar = 10 μm. **(I-M)** Allelism test showing that *Atpch2-1/Atpch2-2* has similar meiotic defects as *Atpch2-1* mutant. Chromosome spread preparations of *Atpch2-1/Atpch2-2* PMCs at late prophase I **(I)**; metaphase I **(J)** and dyad **(K)**. Graph showing the mean silique length **(L)** and mean seed-set per silique **(M)** from 50 siliques of wild type, *Atpch2-1/Atpch2-2* and *Atpch2-1* mutants. Error bars represent the standard deviation. Black stars represent a mean statistical difference between wild type and mutant.
(TIF)

S6 Fig Metaphase I chromosome spreads in *Atmsh5-1* and *Atmsh5-1/Atpch2-1* PMCs. **(A-C)** Chromosome spread preparation of wild type **(A)**, *Atmsh5-1* **(B)** and *Atmsh5-1/Atpch2-1* **(C)** PMCs at metaphase I stage. Chromatin was stained with DAPI (blue) and the chromosomes were labelled with 5S (red) and 45S (green) rDNA FISH probes to facilitate the identification of individual chromosomes. The five bivalents were identified and numbered (white), shown for the wild type nucleus. Bar = 10 μm.
(TIF)

S7 Fig Immunolocalization of axis proteins in wild type and *Atpch2-1*. **(A,B)** Immunolocalization of SYN1 (green) on chromosome spread preparations of wild type **(A)** and *Atpch2-1* mutant **(B)** PMCs. **(C,D)** Immunolocalization of ASY3 (green) on chromosome spread preparations of wild type **(C)** and *Atpch2-1* mutant **(D)** PMCs. DNA is stained with DAPI (blue). Bar = 10 μm.
(TIF)

S8 Fig ASY1 has an off-axis, chromatin associated localization along synapsed chromsomes in wild type PMCs. **(A)** Dual localization of ASY1 (green) and ZYP1 (red) on a chromosome spread of an Arabidopsis PMC at zygotene. **(B)** Localization of ASY1 (green) on chromosome spread of the same meiotic nucleus as **A**. DNA is stained with DAPI (blue). Scale bar = 10 μm. **(C)** shows magnified sections of axes from **A** and **B.** Arrowheads indicate the off-axis, chromatin associated signal of ASY1. Scale bar = 1 μm.
(TIF)

S9 Fig Quantification of axis associated ASY1 in synapsed and unsynapsed regions. **(A)** Example immunostained wild type PMC with a fluorescent microsphere control used for calibration (see Materials and Methods for details); **(B)** Brighter version of **(A)** to highlight the PMC: **(C-F)** Sections of axis (stained as indicated) were analysed for quantification of ASY1 signal intensity. In wild type PMCs **(C,D)**, ASY1 signal intensity is reduced in synapsed chromosome regions (red dotted outline) compared to unsynapsed regions (yellow dotted outline). Chromosomes in *Atpch2-1* PMCs **(E,F)** show no such reduction. Scale bar = 5μm.
(TIF)

S10 Fig Co-localization of PCH2 and ZYP1 in wild type and chromosome axis mutants. **(A-C)** Immunolocalization of ZYP1 (green) and PCH2 (red) in wild type at early zygotene. **(D-F)** Immunolocalization of ZYP1 (green) and PCH2 (red) in an *Atasy1* nucleus at mid-prophase I. **(G-I)** Immunolocalization of ZYP1 (green) and PCH2 (red) in an *Atasy3* nucleus at mid-prophase I. DNA is stained with DAPI (blue). Bar = 10 μm.
(TIF)

S11 Fig PCH2 is not detected in *Atpch2* mutants. **(A-C)** Immunolocalization of PCH2 (red) on chromosome spreads from *Atpch2-1* **(A)**, *Atpch2-2* **(B)** and *Atpch2-3* **(C)** nuclei at mid/late prophase I. DNA is stained with DAPI (blue). Bar = 10 μm.
(TIF)

S12 Fig Immunolocalization of PCH2 in wild type *B*. *oleracea* PMCs. **(A)** Co-localization of ASY1 (green) and PCH2 (red) at late G2 and **(B)** early leptotene using SIM. **(C)** Co-localization of ASY1 (green) and PCH2 (red) at late zygotene using SIM. On synapsed regions (boxed region and corresponding inset which has been brightened for clarity of PCH2 foci and residual ASY1 signal) the ASY1 signal strength is reduced relative to remaining unsynapsed axes (arrowed). **(D)** Co-localization of ZYP1 (green) and PCH2 (red) at pachytene using SIM. DNA is stained with DAPI (blue). Bar = 10 μm.
(TIF)

S13 Fig Prophase I progression is delayed in *Atpch2-1*. **(A)** Comparison of progression through prophase I in wild type and *Atpch2-1* PMCs reveals a delay of 5-8h in the mutant. **(B)** Examples of samples taken at different time points showing the extent of meiotic progression. (Note: as synapsis was incomplete in *Atpch2-1*, fully synapsed pachytene nuclei were not observed).
(TIF)

S14 Fig FTL analysis. **(A)** Map indicating the position on the chromosomes of the genetic markers used to measure the recombination frequency. **(B)** Table showing the location and size of the six FTL genetic intervals used in this study. **(C)** Tetrad pollen expressing the fluorescent proteins were classified into 12 groups based on the distribution of the fluorescent proteins in the tetrad. A schematic representation of the expected patterns of the fluorescent proteins in the tetrad pollen after recombination events is shown for each group.
(TIF)

S15 Fig Co-immunolocalization of ZYP1 and HEI10 in wild type *B*. *oleracea* PMCs at the leptotene/zygotene transition. **A-D** Dual localization of ZYP1 (green) and HEI10 (red) on chromosome spread preparations of *B*. *oleracea* PMCs at the leptotene/zygotene transition. White arrows in **C** and **D** indicate examples of ZYP1 and HEI10 colocalization at SC nucleation sites. DNA is stained with DAPI (blue). Bar = 10 μm.
(TIF)

S1 Table ASY1 intensity measurements on synapsed and unsynapsed regions in wild type and *Atpch2-1* PMCs. (PDF)

S2 Table Tables showing the recombination frequency (f) of the adjacent sets of genetic intervals I5a/b, I5c/d and I2f/g and their coefficient of coincidence (CoC) in wild type and *Atpch2-1*. (TIF)

S3 Table Primer sequences used during this study. **Primer pairs 1–2, 3–4, 5–6 were used to map the T-DNA insertions site of *Atpch2-1*, *Atpch2-2* and *Atpch2-3* respectively.** Primers 4,6–8 were used for the analysis of *AtPCH2* expression. Primers 1,3,5, 9–14 were used for genotyping.
(TIF)
