# Supplementary figures and images for: Arabidopsis PCH2 Mediates Meiotic Chromosome Remodeling and Maturation of Crossovers
Source: PLoS Genet. 2015 Jul 16;11(7):e1005372. doi: 10.1371/journal.pgen.1005372 (PMC4504720; doi:10.1371/journal.pgen.1005372)

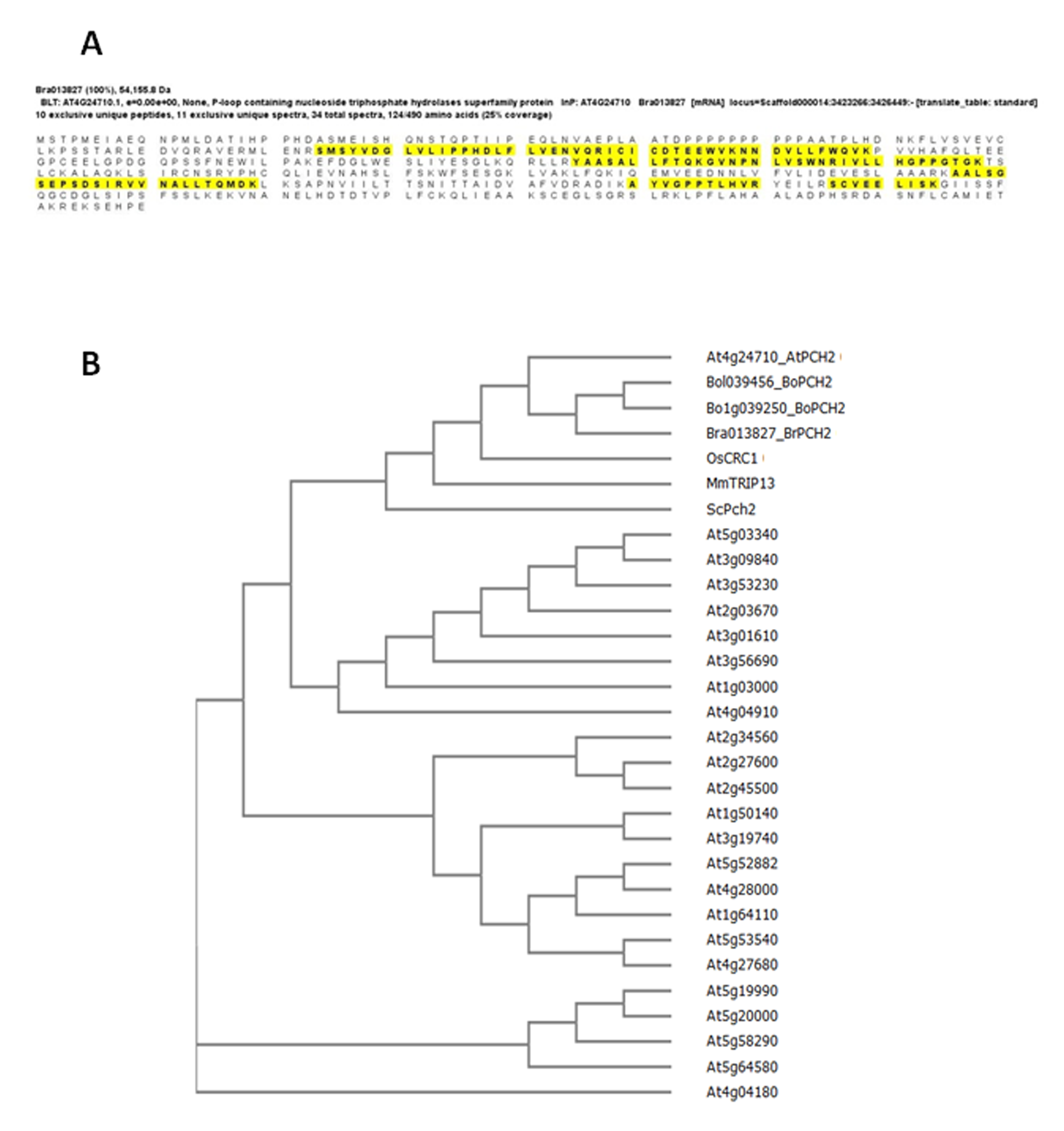

Supplement: S1 Fig — (A) Sequence coverage of the predicted protein product of Bra013827 (yellow highlight). (B) Cladogram derived from ClustalW2 analysis of AAA+ATPase proteins from A. thaliana. At4g24710 belongs to a sub-family which also includes Bra013827 and PCH2 homologues from budding yeast (ScPCH2), mouse (MmTRIP13) and rice (OsCRC1). During the course of this work the sequence of two B. oleracea PCH2 orthologues became available (Liu et al. 2014; Parkin et al. 2014) and are included in the analysis. (TIF) [file pgen.1005372.s001.tif]

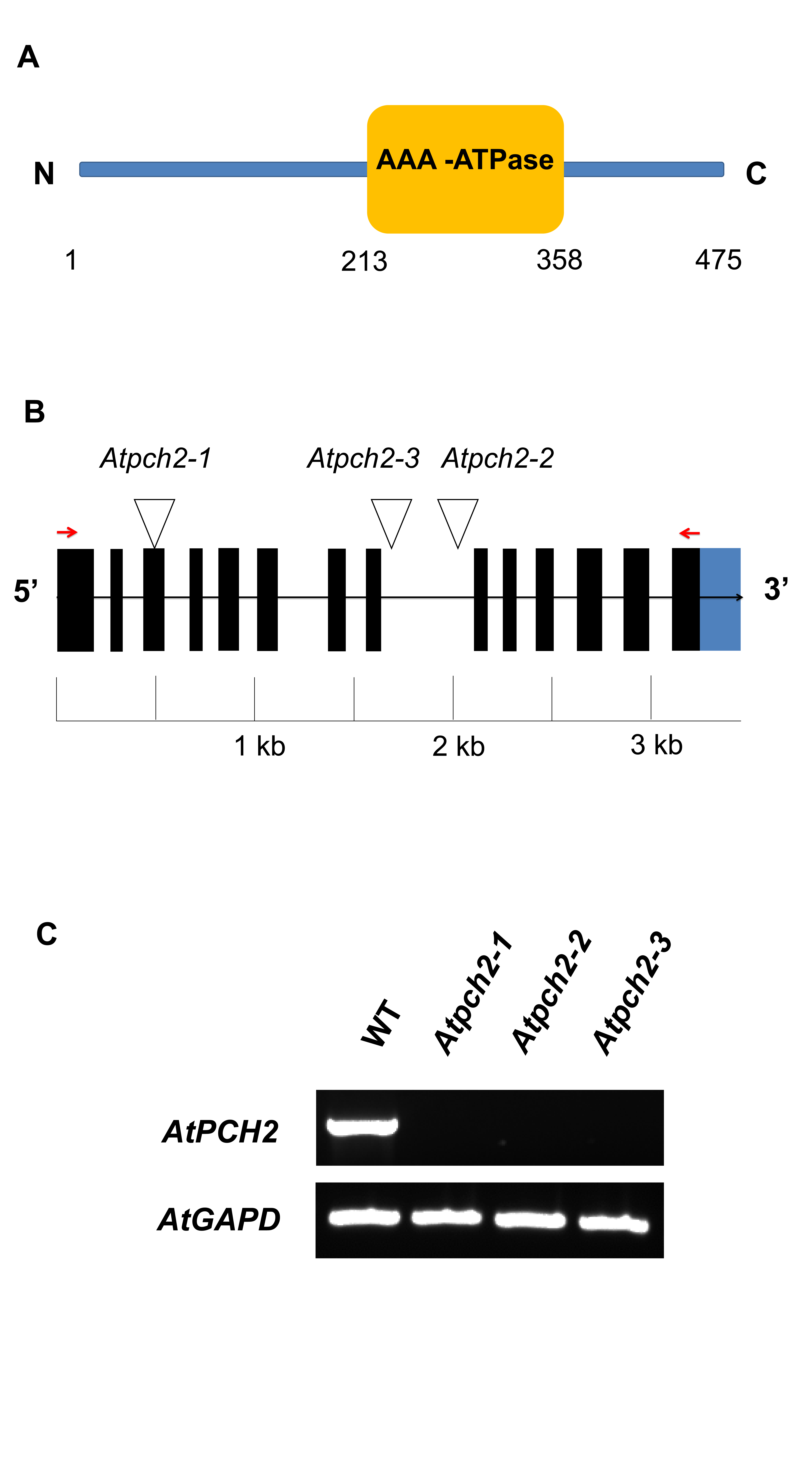

Supplement: S2 Fig — (A) Schematic illustration of Arabidopsis PCH2 protein. AtPCH2 is predicted to encode a protein of 475 amino acids with a putative AAA-ATPase domain located between amino acids 213 and 358. (B) Map of AtPCH2 locus showing the exon/intron organization. Exons are represented with black boxes. 3’ UTR region is represented with a blue box. Triangles represent the location of T-DNA insertion sites for all three Atpch2 mutants. Red arrows mark the position of the primers used for detecting the full-length AtPCH2 transcript by RT-PCR. (C) Gene expression analysis of AtPCH2 using semi-quantitative RT-PCR from wild type and Atpch2-1, Atpch2-2 and Atpch2-3 bud tissues. The amount of RNA used for each sample was equalized using the housekeeping gene AtGAPD. AtPCH2 was expressed in wild type buds while no full length transcript was detected in Atpch2 mutants. (TIF) [file pgen.1005372.s002.tif]

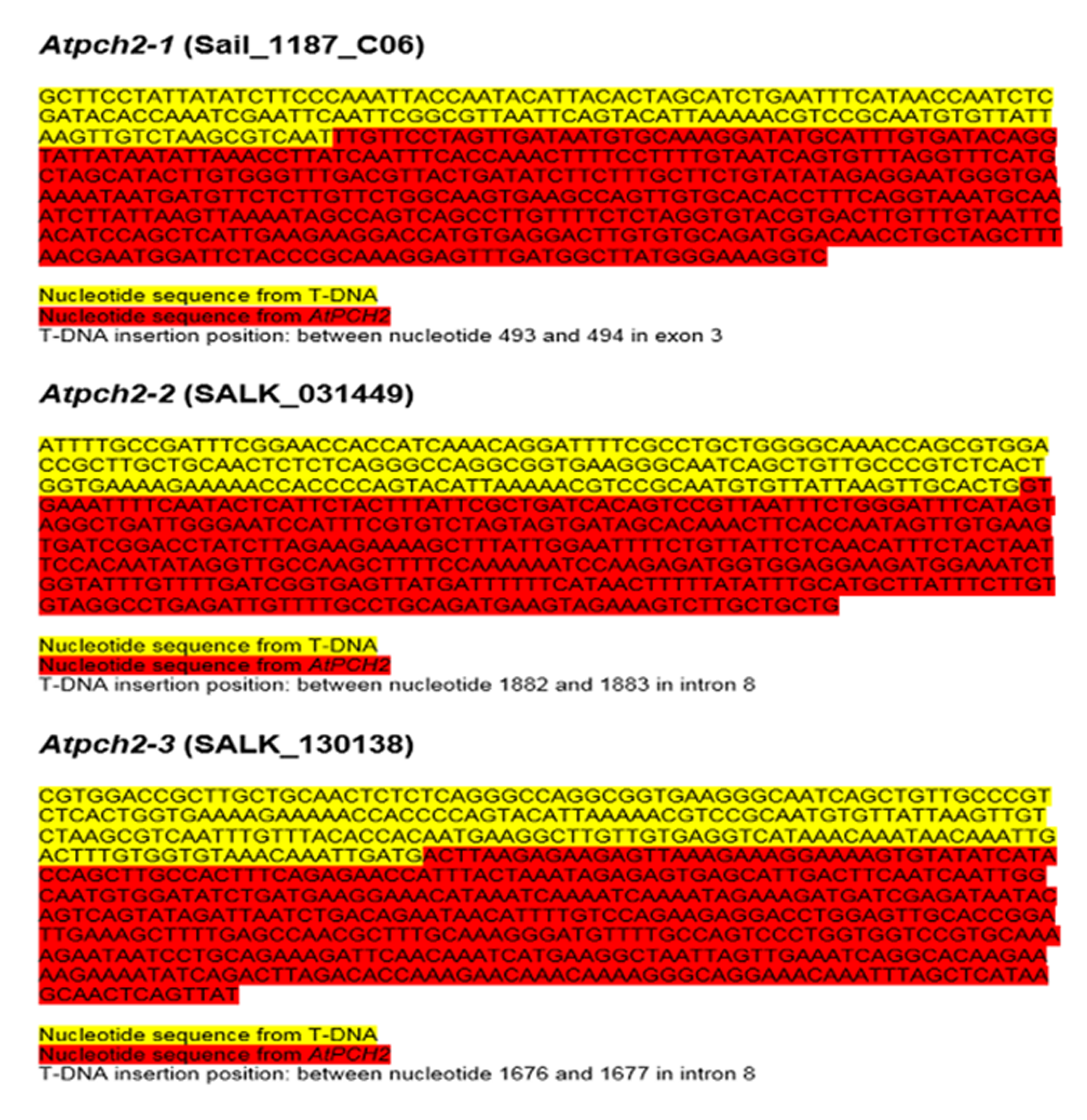

Supplement: S3 Fig — (TIF) [file pgen.1005372.s003.tif]

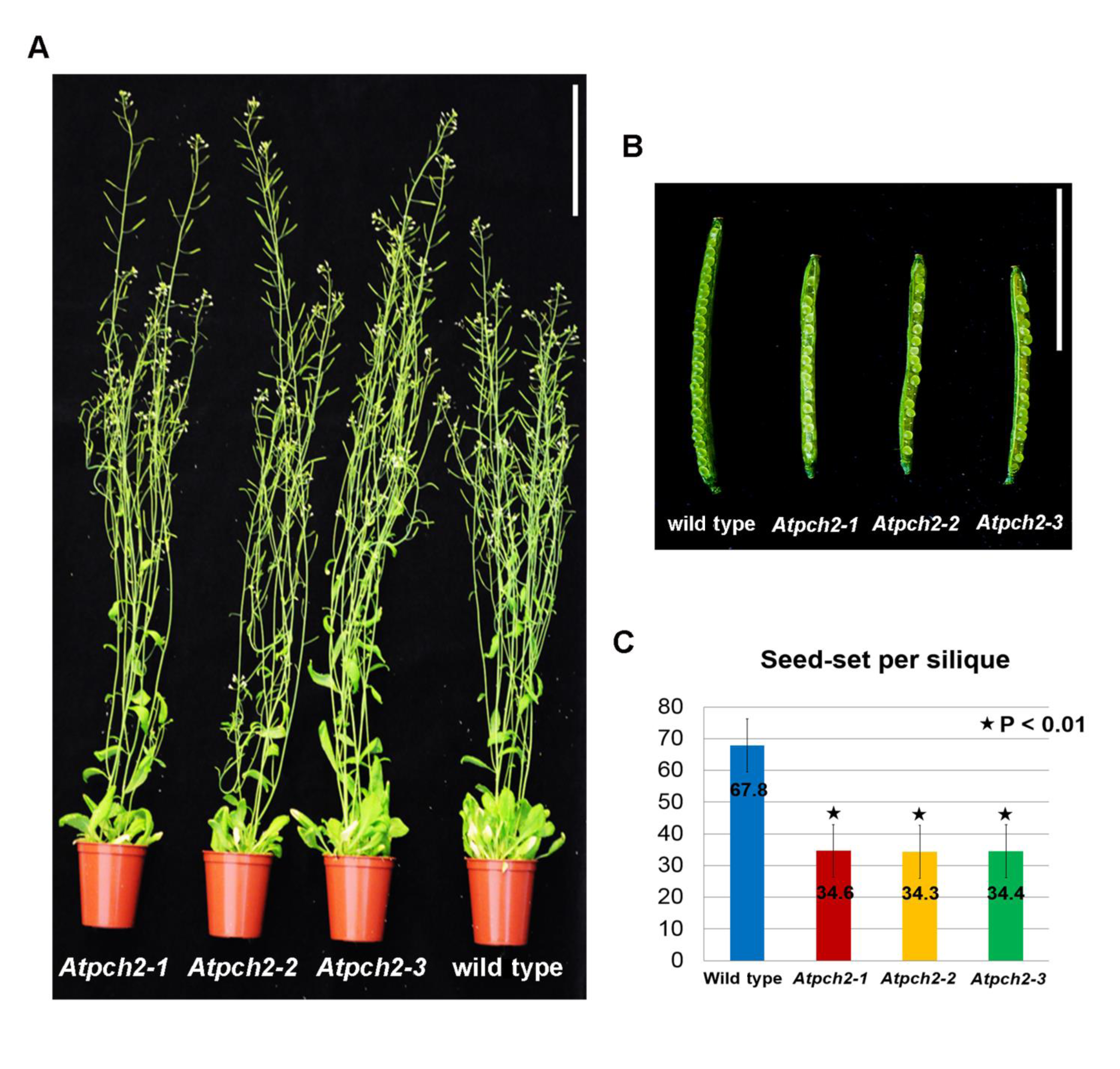

Supplement: S4 Fig — (A) Vegetative growth is normal but fertility is reduced in Atpch2 mutants. Bar = 5 cm. (B) Silique length is slightly reduced and numerous gaps are observed between the seeds in Atpch2 mutants. Bar = 1 cm. (C) Graph showing the mean seed-set per silique from 50 siliques of wild type Arabidopsis and Atpch2 mutants. Error bars represent the standard deviation. Black stars represent a mean statistical difference between wild type and mutant. (TIF) [file pgen.1005372.s004.tif]

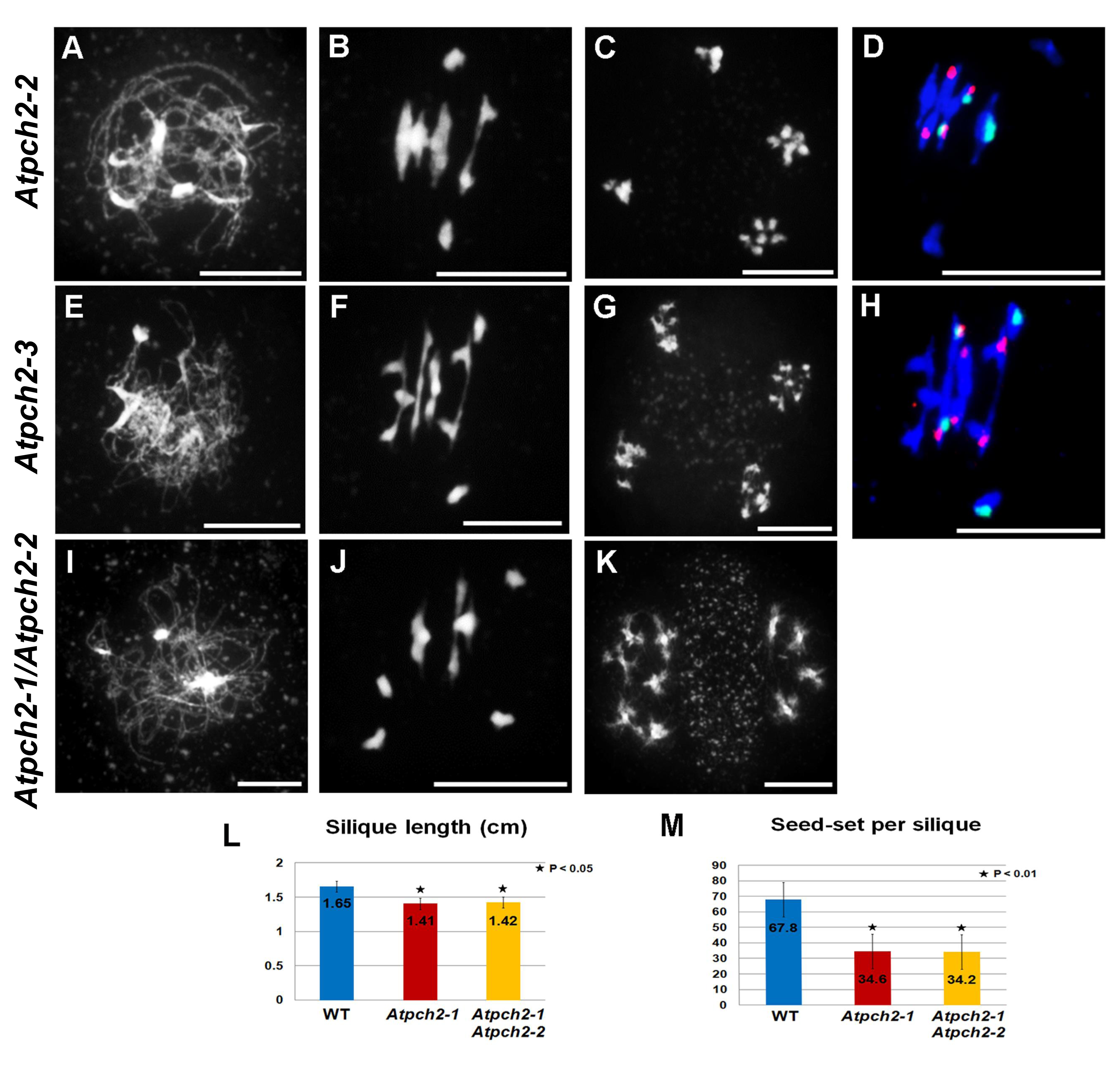

Supplement: S5 Fig — (A-D) Meiotic stages in Atpch2-2 mutant. (A) late prophase I; (B) metaphase I; (C) tetrad; (D) metaphase I nucleus labelled with 5S (red) and 45S (green) rDNA probes. (E-H) Meiotic stages in Atpch2-3 mutant. (E) late prophase I; (F) metaphase I; (G) tetrad; (H) metaphase I nucleus labelled with 5S (red) and 45S (green) rDNA probes. Bar = 10 μm. (I-M) Allelism test showing that Atpch2-1/Atpch2-2 has similar meiotic defects as Atpch2-1 mutant. Chromosome spread preparations of Atpch2-1/Atpch2-2 PMCs at late prophase I (I); metaphase I (J) and dyad (K). Graph showing the mean silique length (L) and mean seed-set per silique (M) from 50 siliques of wild type, Atpch2-1/Atpch2-2 and Atpch2-1 mutants. Error bars represent the standard deviation. Black stars represent a mean statistical difference between wild type and mutant. (TIF) [file pgen.1005372.s005.tif]

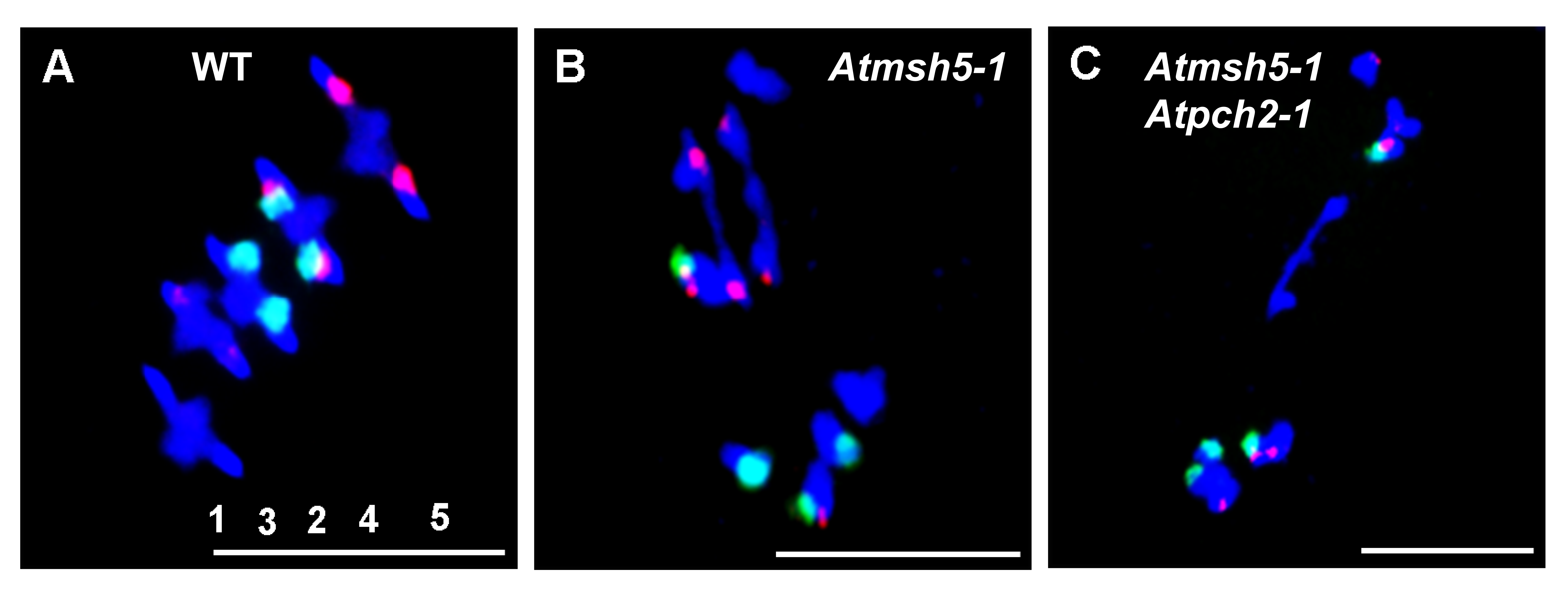

Supplement: S6 Fig — (A-C) Chromosome spread preparation of wild type (A), Atmsh5-1 (B) and Atmsh5-1/Atpch2-1 (C) PMCs at metaphase I stage. Chromatin was stained with DAPI (blue) and the chromosomes were labelled with 5S (red) and 45S (green) rDNA FISH probes to facilitate the identification of individual chromosomes. The five bivalents were identified and numbered (white), shown for the wild type nucleus. Bar = 10 μm. (TIF) [file pgen.1005372.s006.tif]

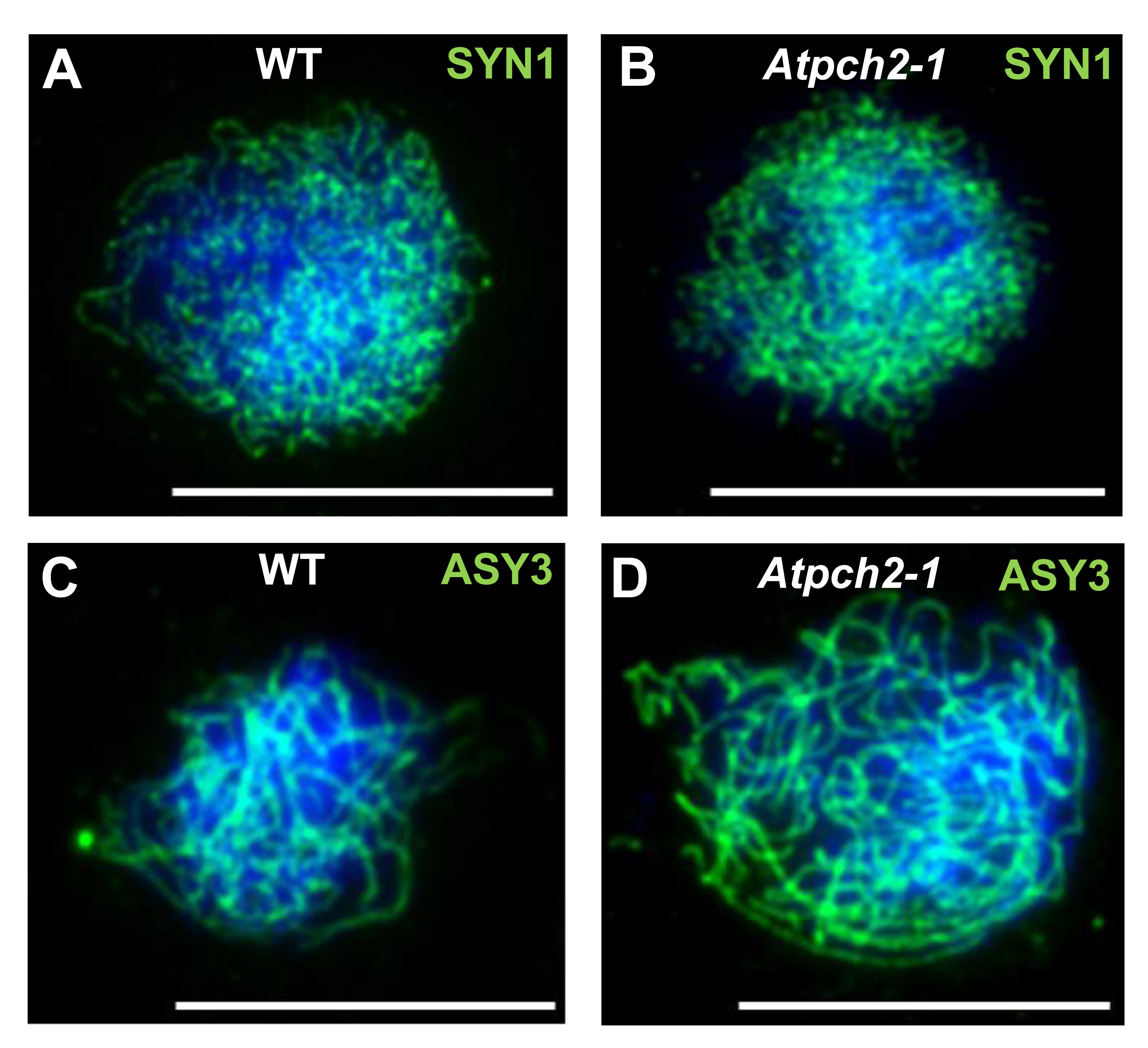

Supplement: S7 Fig — (A,B) Immunolocalization of SYN1 (green) on chromosome spread preparations of wild type (A) and Atpch2-1 mutant (B) PMCs. (C,D) Immunolocalization of ASY3 (green) on chromosome spread preparations of wild type (C) and Atpch2-1 mutant (D) PMCs. DNA is stained with DAPI (blue). Bar = 10 μm. (TIF) [file pgen.1005372.s007.tif]

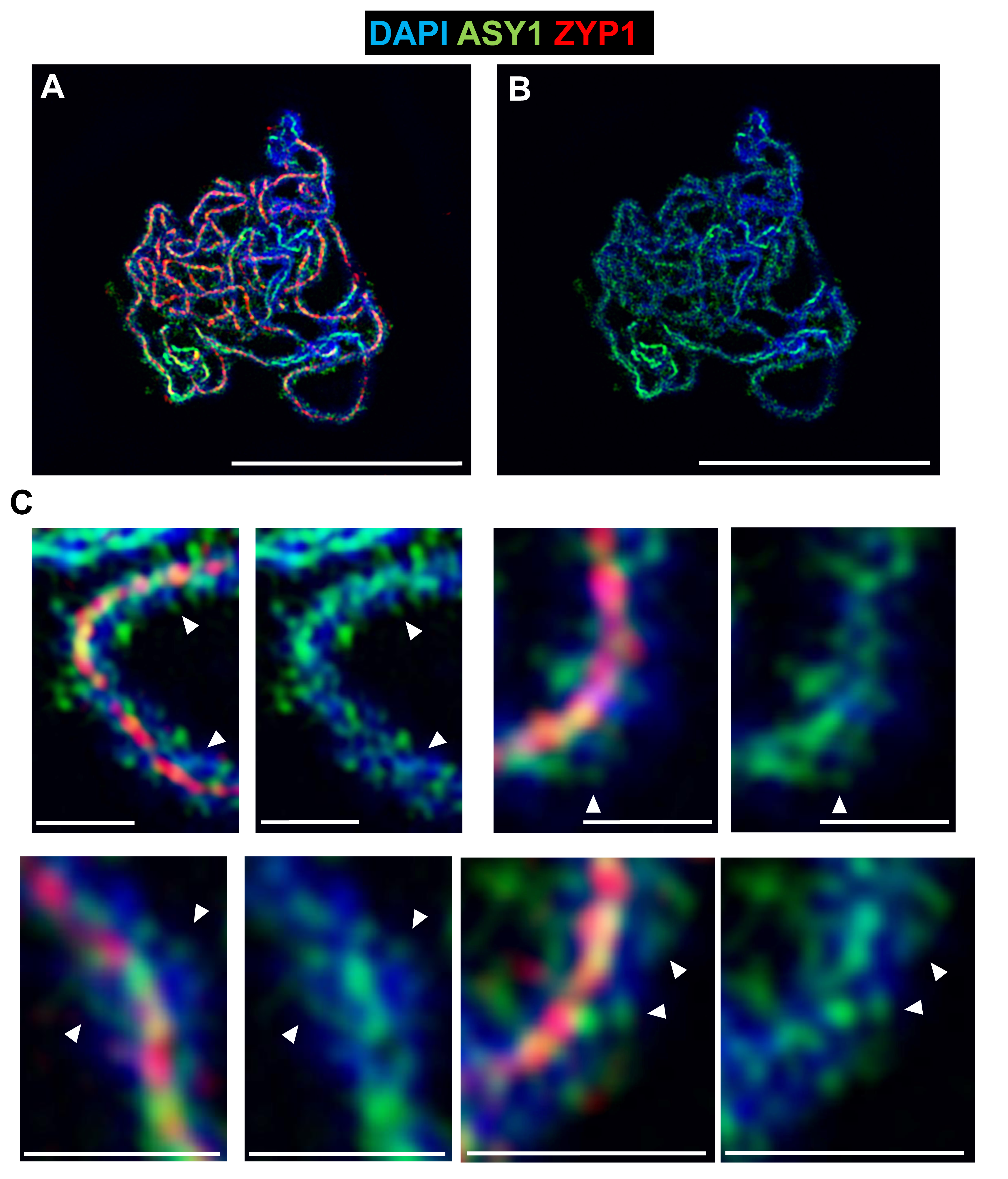

Supplement: S8 Fig — (A) Dual localization of ASY1 (green) and ZYP1 (red) on a chromosome spread of an Arabidopsis PMC at zygotene. (B) Localization of ASY1 (green) on chromosome spread of the same meiotic nucleus as A. DNA is stained with DAPI (blue). Scale bar = 10 μm. (C) shows magnified sections of axes from A and B. Arrowheads indicate the off-axis, chromatin associated signal of ASY1. Scale bar = 1 μm. (TIF) [file pgen.1005372.s008.tif]

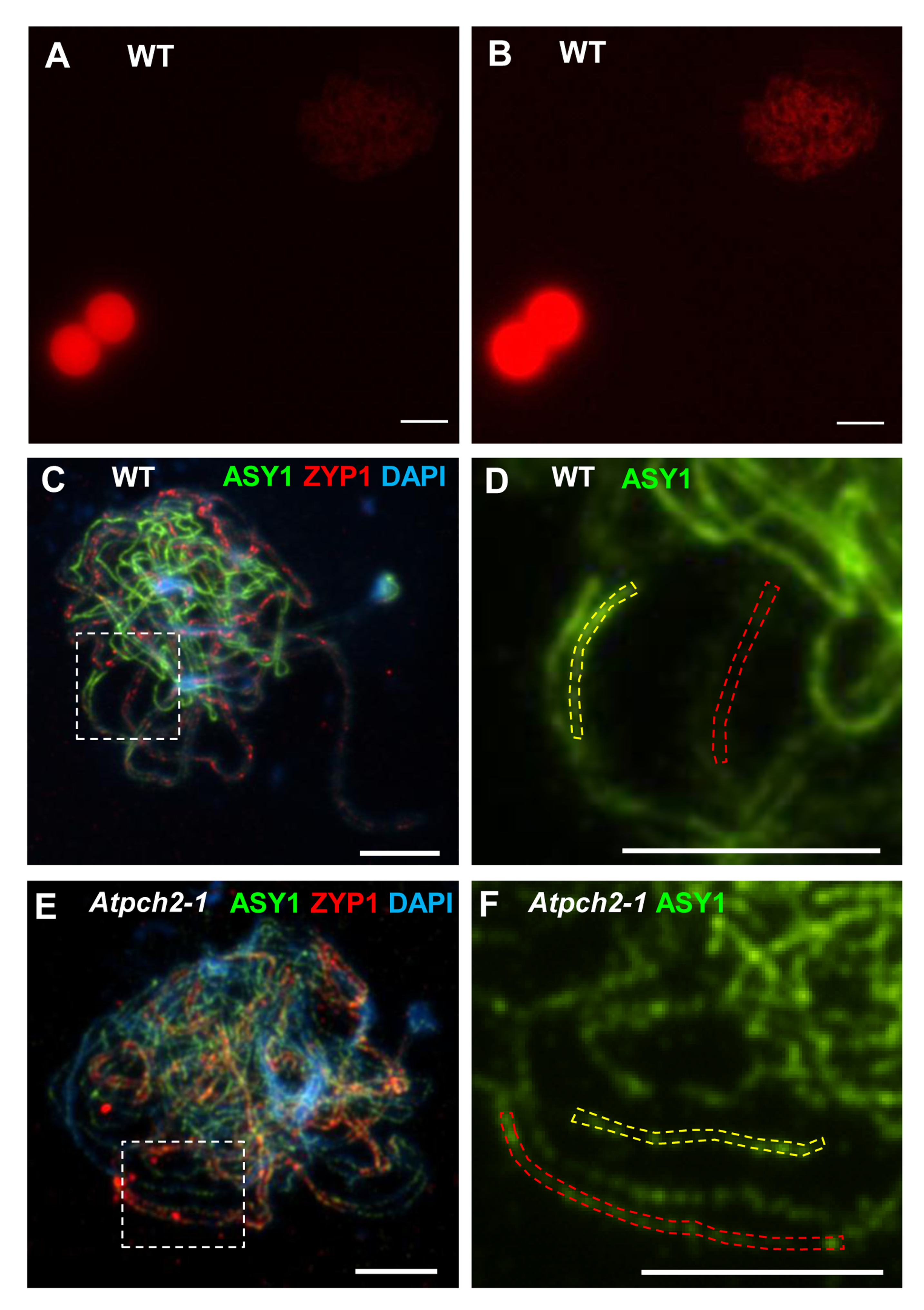

Supplement: S9 Fig — (A) Example immunostained wild type PMC with a fluorescent microsphere control used for calibration (see Materials and Methods for details); (B) Brighter version of (A) to highlight the PMC: (C-F) Sections of axis (stained as indicated) were analysed for quantification of ASY1 signal intensity. In wild type PMCs (C,D), ASY1 signal intensity is reduced in synapsed chromosome regions (red dotted outline) compared to unsynapsed regions (yellow dotted outline). Chromosomes in Atpch2-1 PMCs (E,F) show no such reduction. Scale bar = 5μm. (TIF) [file pgen.1005372.s009.tif]

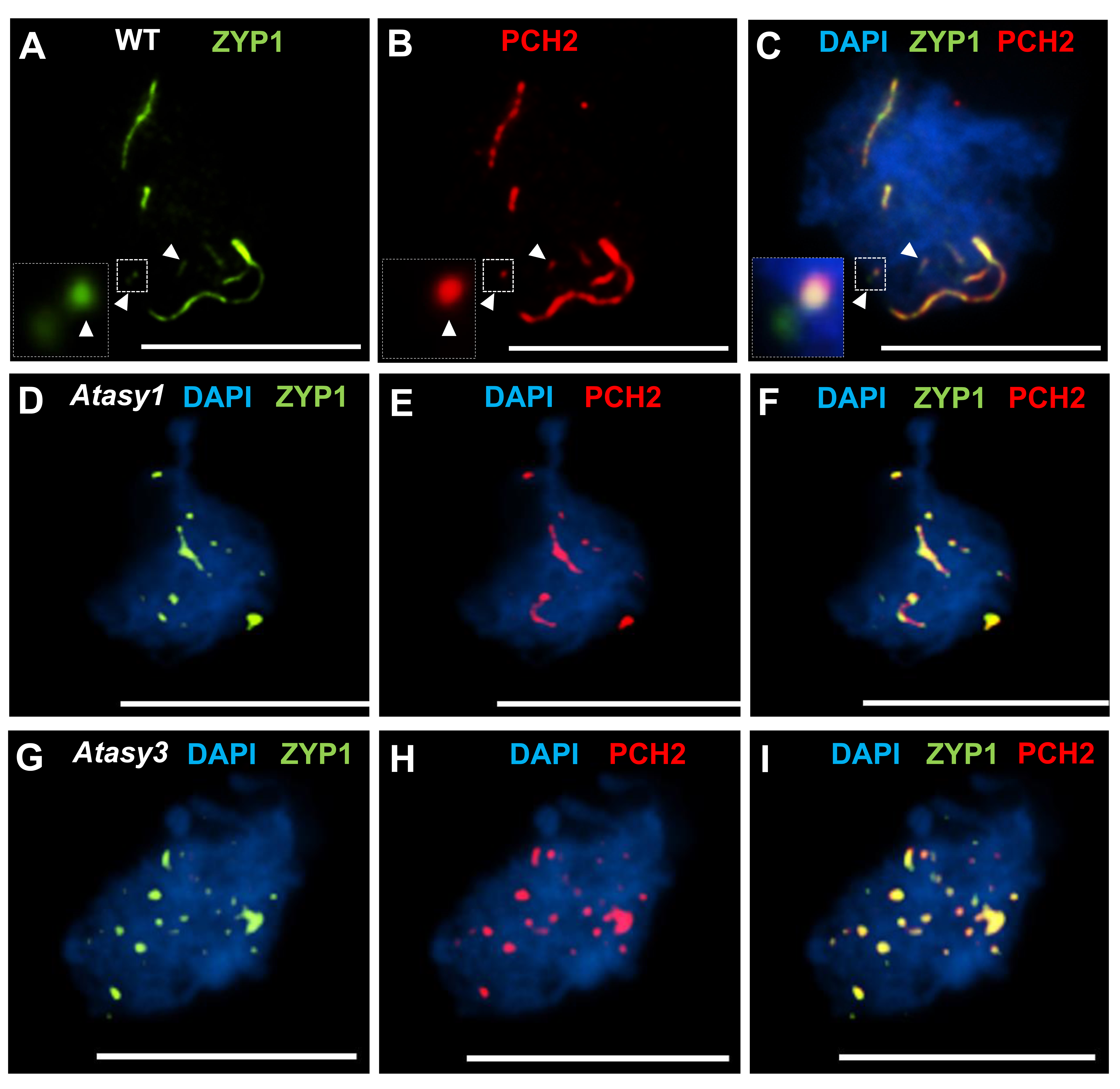

Supplement: S10 Fig — (A-C) Immunolocalization of ZYP1 (green) and PCH2 (red) in wild type at early zygotene. (D-F) Immunolocalization of ZYP1 (green) and PCH2 (red) in an Atasy1 nucleus at mid-prophase I. (G-I) Immunolocalization of ZYP1 (green) and PCH2 (red) in an Atasy3 nucleus at mid-prophase I. DNA is stained with DAPI (blue). Bar = 10 μm. (TIF) [file pgen.1005372.s010.tif]

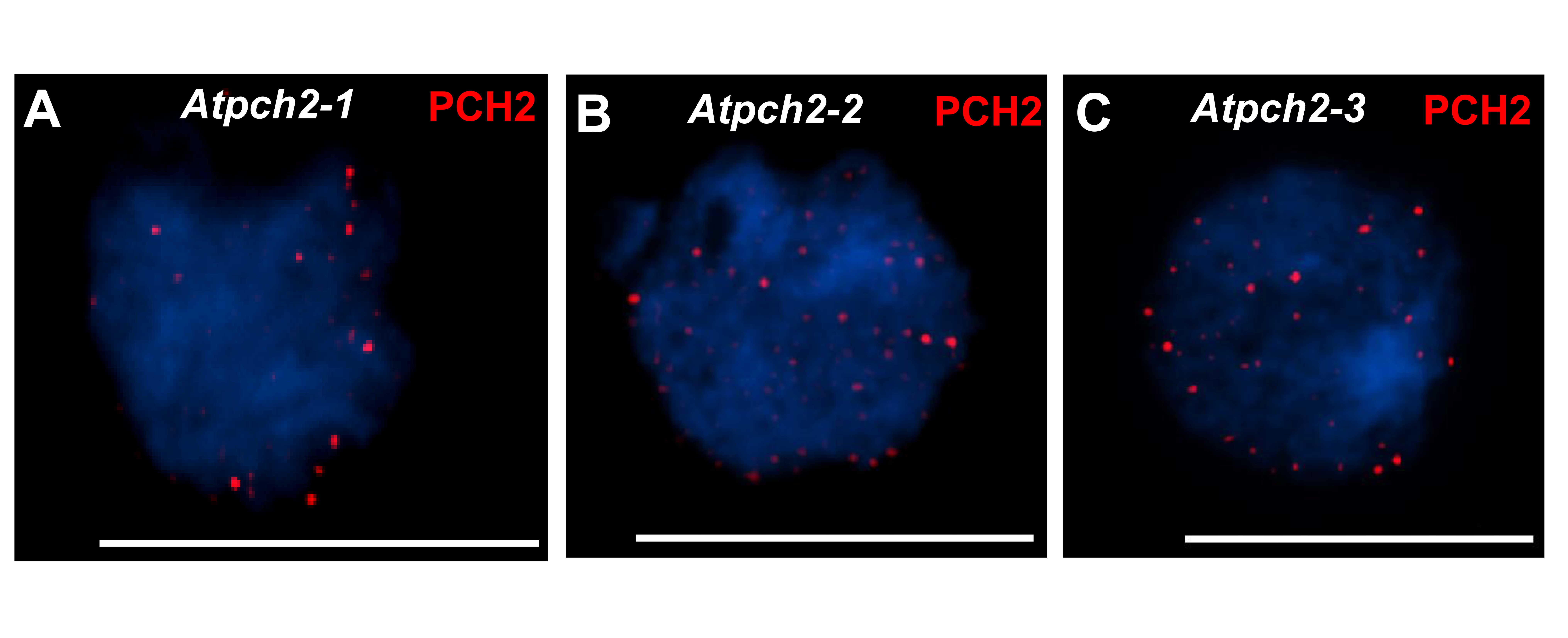

Supplement: S11 Fig — (A-C) Immunolocalization of PCH2 (red) on chromosome spreads from Atpch2-1 (A), Atpch2-2 (B) and Atpch2-3 (C) nuclei at mid/late prophase I. DNA is stained with DAPI (blue). Bar = 10 μm. (TIF) [file pgen.1005372.s011.tif]

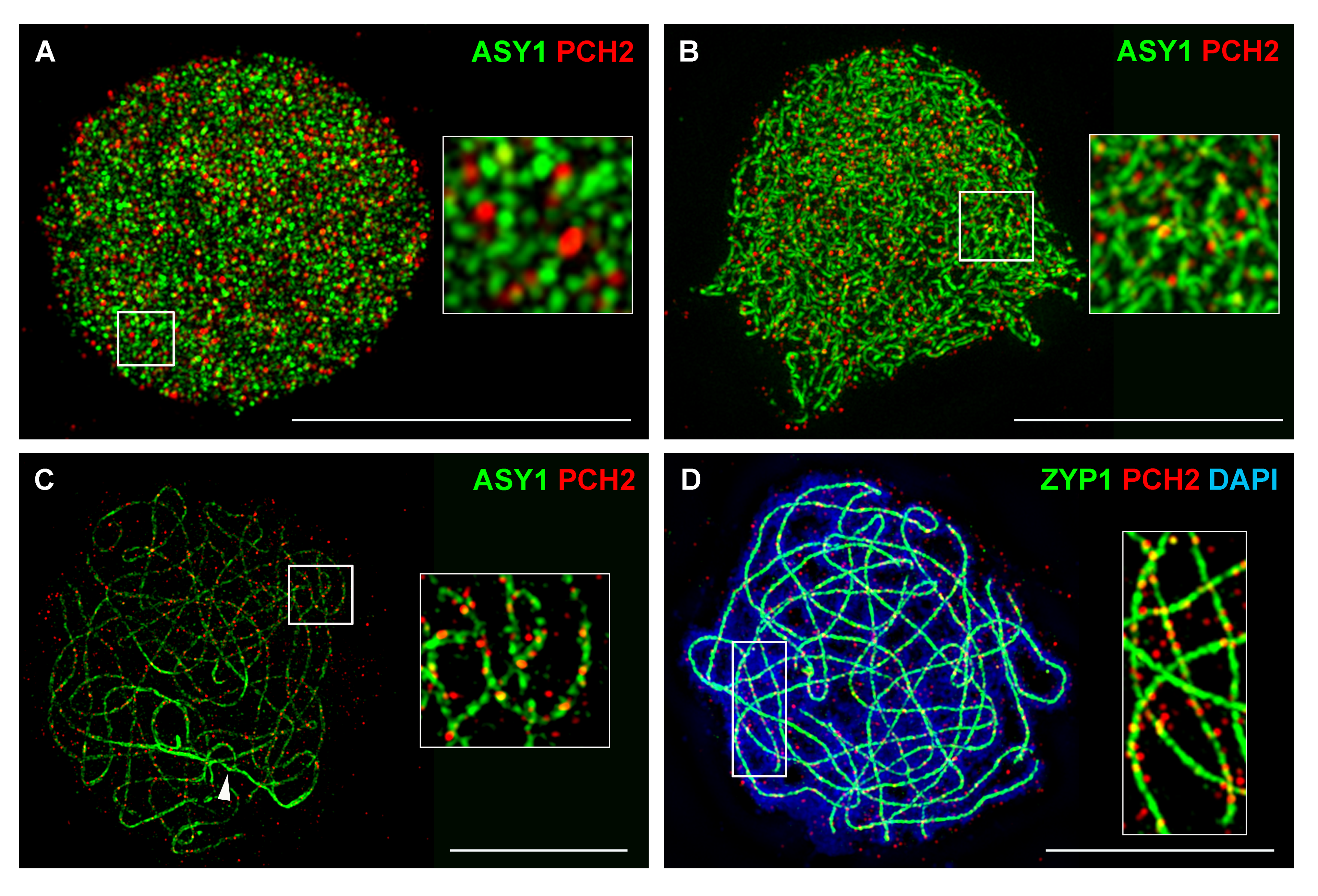

Supplement: S12 Fig — (A) Co-localization of ASY1 (green) and PCH2 (red) at late G2 and (B) early leptotene using SIM. (C) Co-localization of ASY1 (green) and PCH2 (red) at late zygotene using SIM. On synapsed regions (boxed region and corresponding inset which has been brightened for clarity of PCH2 foci and residual ASY1 signal) the ASY1 signal strength is reduced relative to remaining unsynapsed axes (arrowed). (D) Co-localization of ZYP1 (green) and PCH2 (red) at pachytene using SIM. DNA is stained with DAPI (blue). Bar = 10 μm. (TIF) [file pgen.1005372.s012.tif]

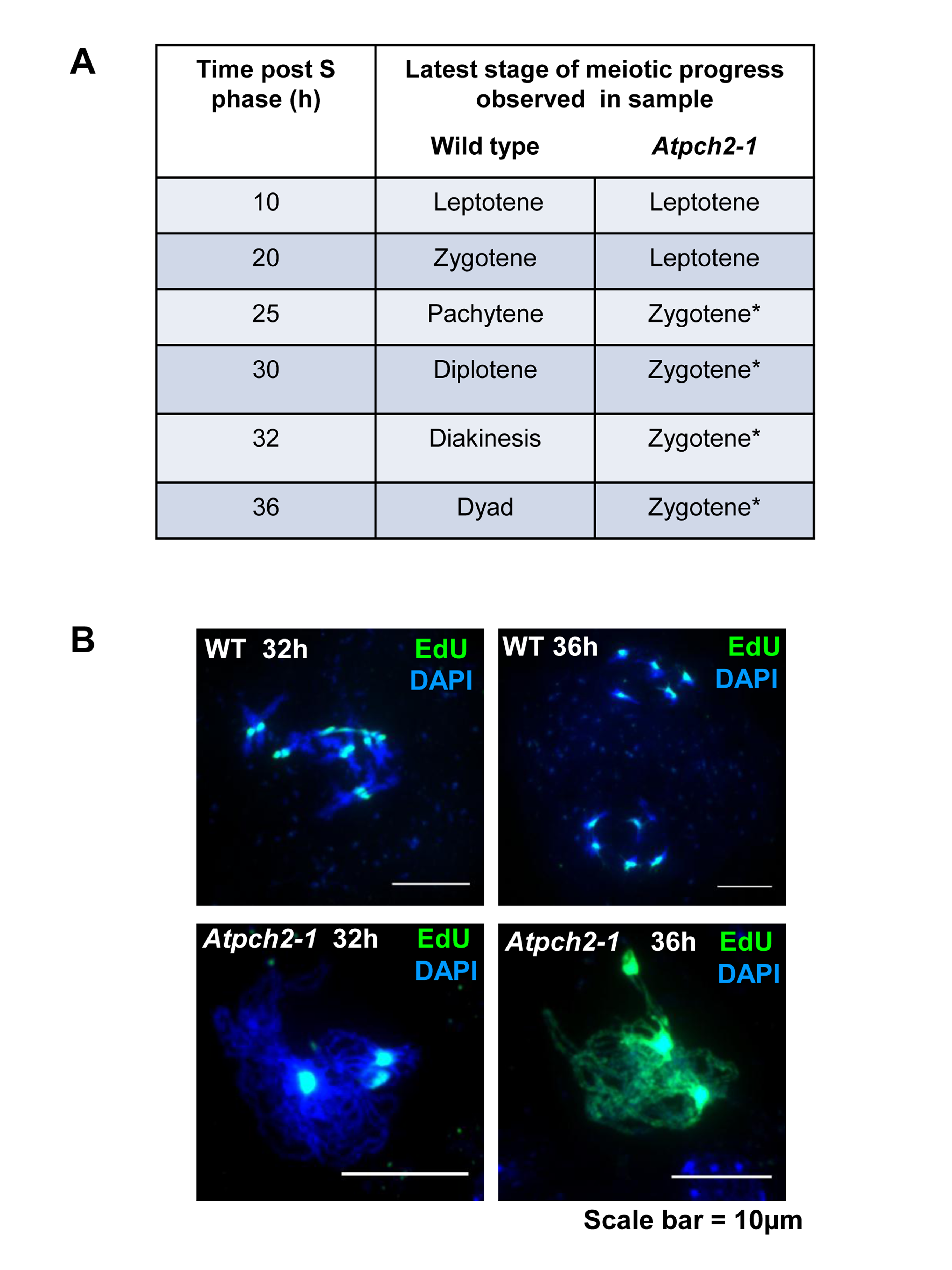

Supplement: S13 Fig — (A) Comparison of progression through prophase I in wild type and Atpch2-1 PMCs reveals a delay of 5-8h in the mutant. (B) Examples of samples taken at different time points showing the extent of meiotic progression. (Note: as synapsis was incomplete in Atpch2-1, fully synapsed pachytene nuclei were not observed). (TIF) [file pgen.1005372.s013.tif]

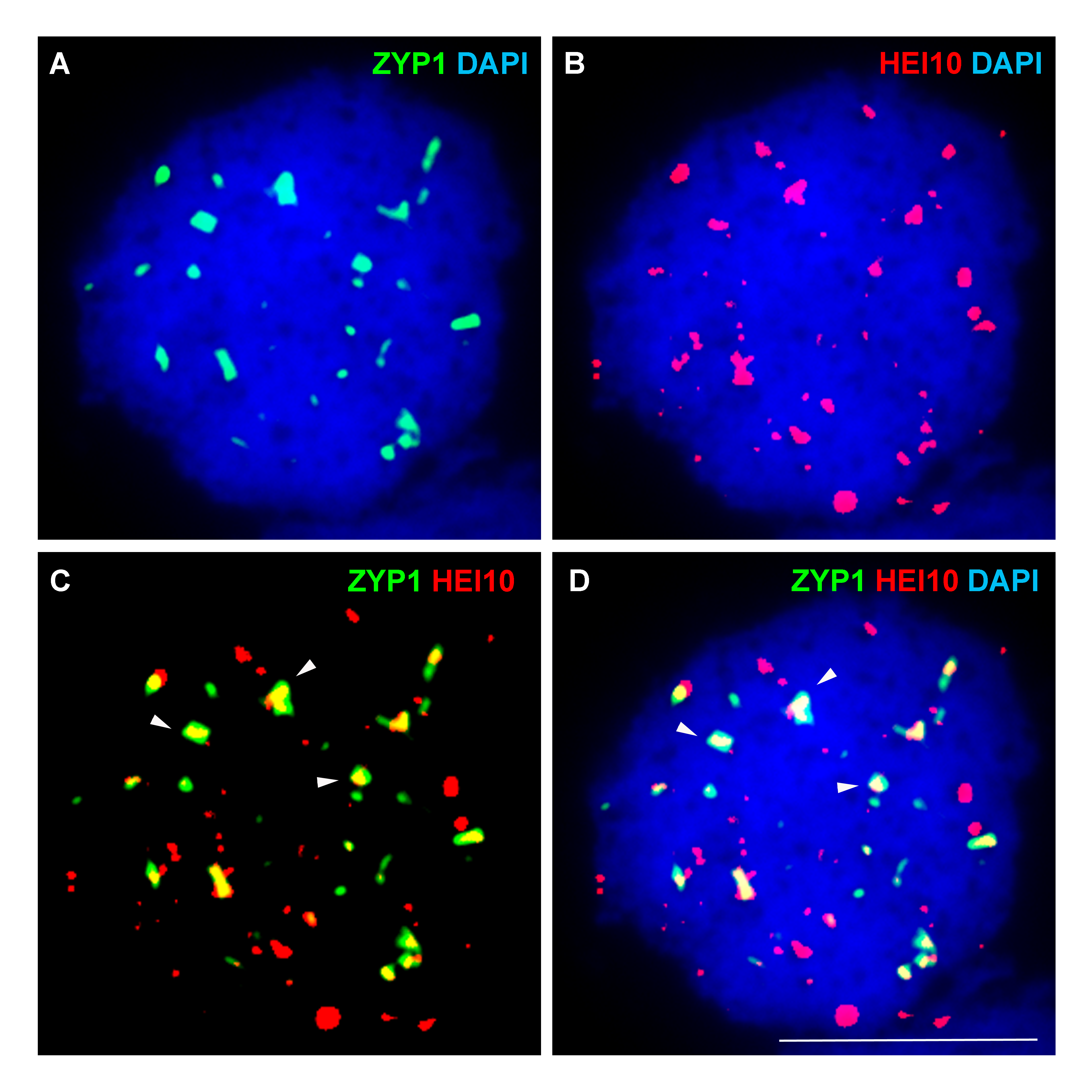

Supplement: S15 Fig — A-D Dual localization of ZYP1 (green) and HEI10 (red) on chromosome spread preparations of B. oleracea PMCs at the leptotene/zygotene transition. White arrows in C and D indicate examples of ZYP1 and HEI10 colocalization at SC nucleation sites. DNA is stained with DAPI (blue). Bar = 10 μm. (TIF) [file pgen.1005372.s015.tif]

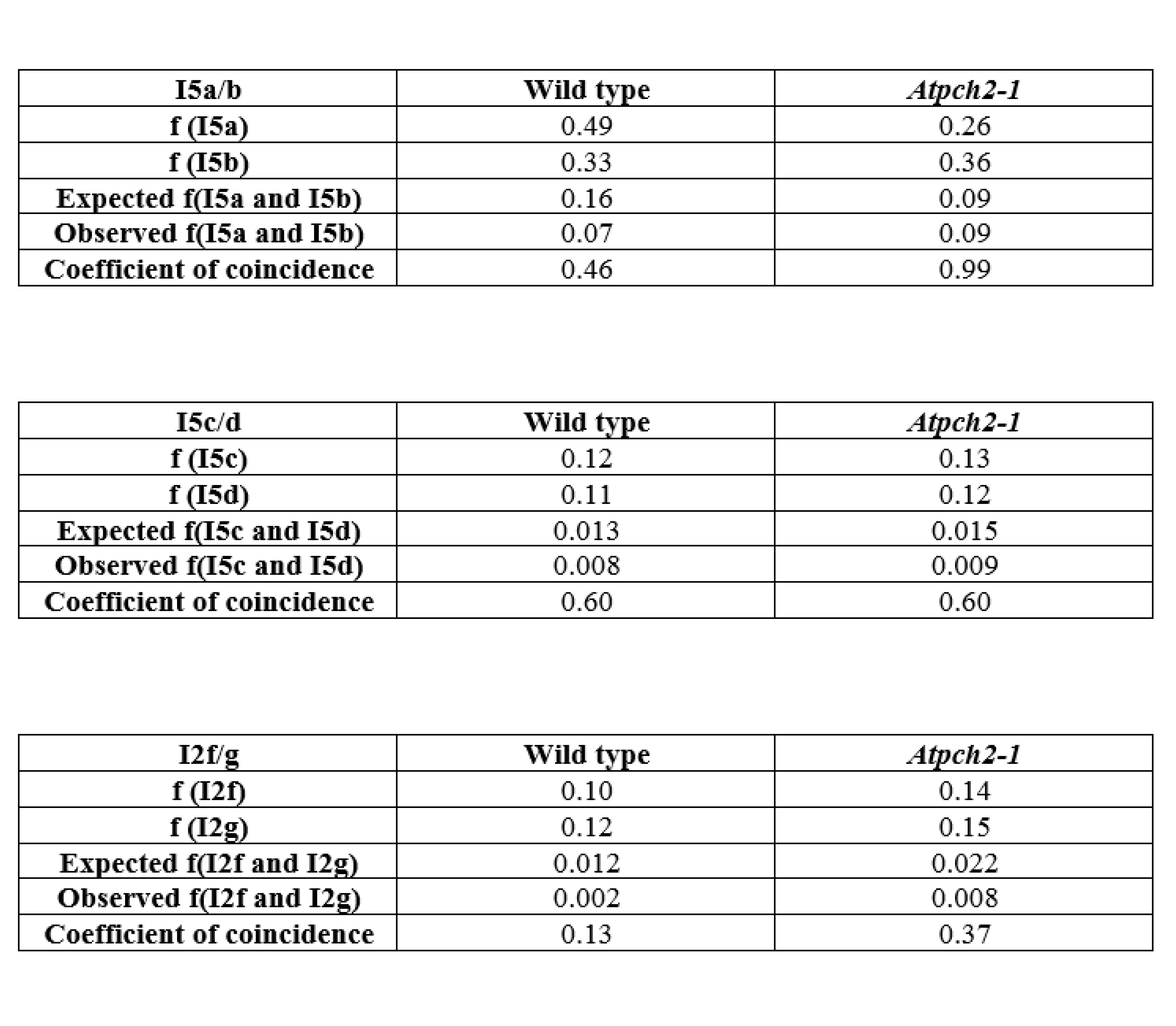

Supplement: S2 Table — (TIF) [file pgen.1005372.s017.tif]

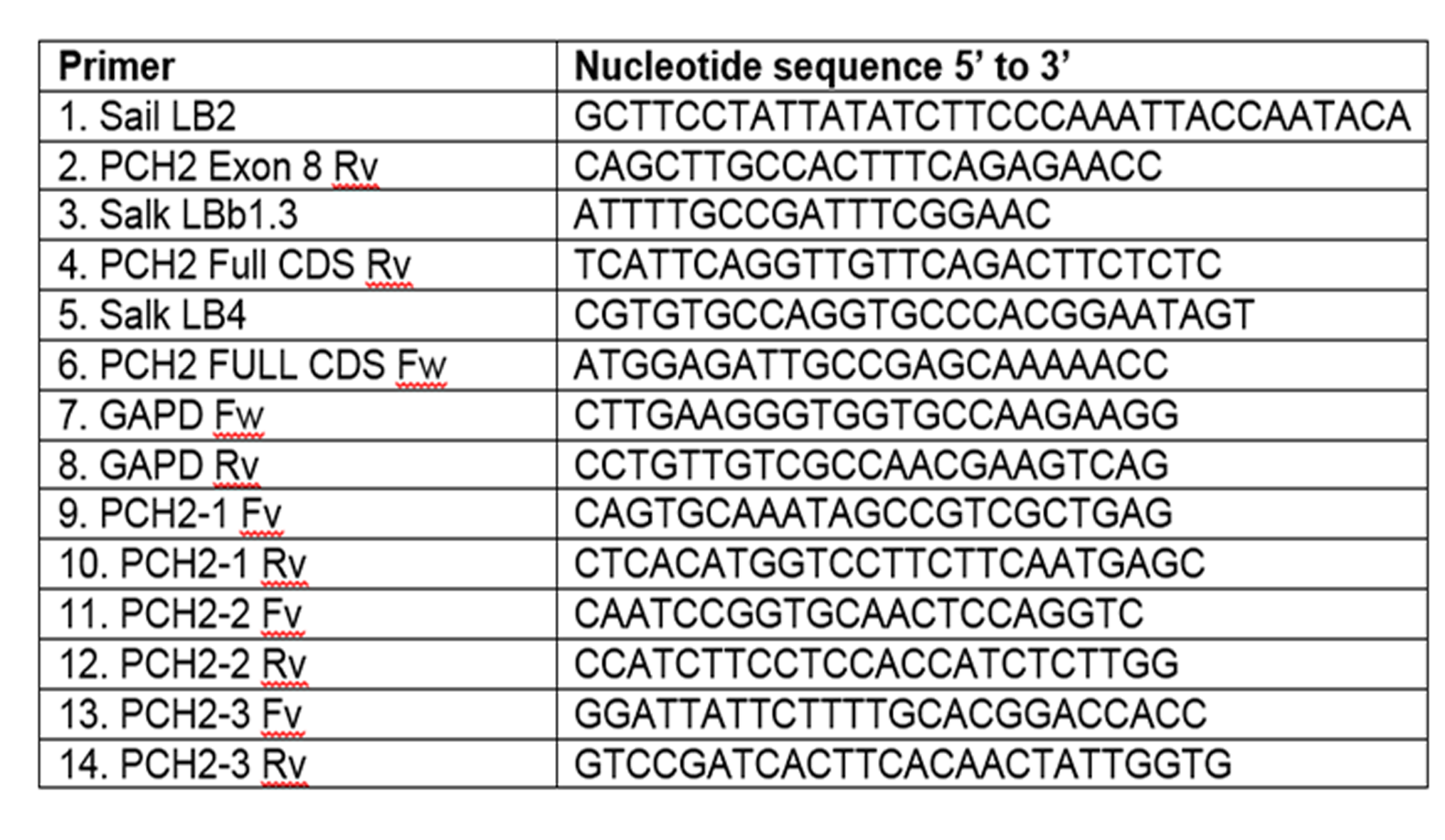

Supplement: S3 Table — Primer pairs 1–2, 3–4, 5–6 were used to map the T-DNA insertions site of Atpch2-1, Atpch2-2 and Atpch2-3 respectively. Primers 4,6–8 were used for the analysis of AtPCH2 expression. Primers 1,3,5, 9–14 were used for genotyping. (TIF) [file pgen.1005372.s018.tif]
