# Supplementary material for: Arabidopsis PCH2 Mediates Meiotic Chromosome Remodeling and Maturation of Crossovers
Source: PLoS Genet. 2015 Jul 16;11(7):e1005372. doi: 10.1371/journal.pgen.1005372 (PMC4504720; doi:10.1371/journal.pgen.1005372)
Supplement: S1 Table — (PDF) [file pgen.1005372.s016.pdf]

Col axis vs SC

Source	RoiID	ROIArea	MeasuredArea	Perimeter	MeanIntensity	MinIntensity	MaxIntensity
slide 2 3_crop roi.nd2	1	0.24	698.22	3.07	582.51	392	911
slide 2 3_crop roi.nd2	3	0.35	698.22	3.61	280.69	213	352
slide 2 3_crop roi.nd2	4	0.45	698.22	3.61	247.97	201	328
10_crop roi.nd2	3	0.46	5452.92	4.47	418.54	272	573
7_crop roi.nd2	1	0.32	1358.79	4.48	1070.84	573	1716
slide 2 3_crop roi.nd2	2	0.38	698.22	4.5	593.65	296	801
10_crop roi.nd2	7	0.52	5452.92	4.55	224.67	158	372
10_crop roi.nd2	9	0.4	5452.92	4.81	1208.53	759	1689
8_crop roi.nd2	5	0.56	744.37	4.92	426.06	371	503
10_crop roi.nd2	1	0.4	5452.92	4.96	830.54	360	1256
7_crop roi.nd2	3	0.57	1358.79	5	426.68	316	616
10_crop roi.nd2	8	0.51	5452.92	5.03	233.07	165	368
8_crop roi.nd2	6	0.77	744.37	6.35	516.59	390	662
7_crop roi.nd2	4	0.71	1358.79	5.25	396.39	278	564
10_crop roi.nd2	2	0.47	5452.92	5.79	902.1	413	1607
7_crop roi.nd2	2	0.38	1358.79	5.89	1199.68	766	1683
10_crop roi.nd2	4	0.48	5452.92	5.9	318.09	212	477
10_crop roi.nd2	6	0.59	5452.92	6.13	619.59	318	1068
8_crop roi.nd2	2	0.48	744.37	6.48	1572.28	1104	2144
8_crop roi.nd2	1	0.84	744.37	7.75	761.16	463	1215
10_crop roi.nd2	11	1.14	5452.92	8.55	217.96	141	350
10_crop roi.nd2	5	0.98	5452.92	9.3	553.36	277	912
10_crop roi.nd2	12	2.19	5452.92	10.52	209.19	132	326
10_crop roi.nd2	10	0.96	5452.92	11.67	856.65	362	1414
slide 3 8_crop roi.nd2	1	1	1516.08	12.01	648.56	329	1080
slide 3 8_crop roi.nd2	2	1.72	1516.08	12.22	155.84	114	225
slide 4 13a_crop	2	0.37	781.5	3.9	543.85	398	681
slide 4 14_crop	2	0.31	1756.41	3.94	437.82	336	584
slide 4 19_crop	2	0.39	1580.05	4.26	588.33	478	734
slide 4 12_crop	1	0.37	898.56	4.69	1993.49	1355	2909

slide 4 17_crop	2	0.54	2388.22	4.76	403.93	315	561
slide 4 18_crop	2	0.36	1082.33	4.92	346.31	244	417
slide 4 13a_crop	1	0.4	781.5	4.93	1016.34	559	1371
slide 4 13b_crop	2	0.61	696.8	5.23	374.31	256	522
slide 4 19_crop	1	0.49	1580.05	5.57	1414.14	826	2206
slide 4 12_crop	2	0.82	898.56	5.62	492.83	385	677
slide 4 16_crop	2	0.66	1589.8	5.7	305.95	202	436
slide 4 15_crop	2	0.54	1872.5	5.78	371.73	262	507
slide 4 13b_crop	1	0.5	696.8	5.81	1490.56	786	2485
slide 4 14_crop	1	0.49	1756.41	5.83	1176.63	792	1541
slide 4 11_crop	2	0.69	643.41	5.92	257.56	213	324
slide 4 18_crop	1	0.54	1082.33	6.72	2045.42	1493	2765
slide 4 17_crop	1	0.79	2388.22	8.18	1079.87	585	1598
slide 4 16_crop	1	0.58	1589.8	8.22	1581.99	933	2296
slide 4 11_crop	1	0.77	643.41	8.82	543.05	366	889
slide 4 15_crop	1	0.93	1872.5	10.4	1086.09	480	2053

**Unsynapsed mean**	1079.44
**Synapsed mean**	356.28

	**Mean ASY1 intensity- bead corrected**					
	Unsynapsed axial elements	Synapsed lateral elements	sections/samples (total)	Number of cells	Bead mean intensity	Number of beads
**Col**	1079.44	356.28	23	17	697.32	11

Col axis vs SC $p<0.00001$ 2 tail, paired t test

Col microspheres

Source	RoiID	ROIArea	MeasuredArea	Perimeter	MeanIntensity	MinIntensity	MaxIntensity
3.nd2	1	20.34	252.61	15.96	707.89	527	1344
12.nd2	1	20.31	380.63	15.96	848.42	650	1225
12.nd2	2	20.14	380.63	15.96	841.69	623	1139
6.nd2	1	21.74	1904.27	16.45	690.78	519	1425
11.nd2	1	23.05	5452.92	17.14	611.37	463	1098
6.nd2	2	22.99	1904.27	17.14	695.2	514	1245
5.nd2	1	23.35	429.52	17.14	686.33	521	1074
5.nd2	2	23.35	429.52	17.14	677.52	512	1092
11.nd2	2	24.2	5452.92	17.55	615.09	484	1012
4.nd2	1	27.51	650.14	18.46	641.37	445	1135
4.nd2	2	27.51	650.14	18.46	654.87	448	1249

**Mean intensity**	697.32

pch2-1 axis vs SC

Source	RoiID	ROIArea	MeasuredArea	Perimeter	MeanIntensity	MinIntensity	MaxIntensity
9_crop roi.nd2	4	0.2	1007.95	2.25	388.45	274	507
8_crop roi.nd2	4	0.18	563.29	3	433.39	359	500
11_crop roi.nd2	4	0.32	1532.5	3.45	235.45	172	305
23_crop roi.nd2	2	0.28	854.18	3.89	435.34	309	715
11_crop roi.nd2	3	0.35	1532.5	3.9	318.64	233	417
8_crop roi.nd2	2	0.31	563.29	4.63	378	282	564
14_crop roi.nd2	4	0.33	1029.75	4.65	321.47	173	537
29_crop roi.nd2	2	0.45	801.68	4.78	373.95	250	486
27_crop roi.nd2	1	0.48	606	5.4	296.36	242	397
27_crop roi.nd2	2	0.52	606	5.54	282.6	226	358
8_crop roi.nd2	3	0.46	563.29	5.56	284.93	199	401
8_crop roi.nd2	1	0.41	563.29	5.79	337.73	207	533
15_crop roi.nd2	1	0.57	1529.35	5.8	245.76	144	416
15_crop roi.nd2	3	0.47	1529.35	5.8	286.54	157	476
16_crop roi.nd2	3	0.59	2790.27	5.83	239.01	165	330
11_crop roi.nd2	1	0.52	1532.5	6.01	287.19	164	424
30_crop roi.nd2	2	0.68	1530.2	6.02	291.97	193	504
11_crop roi.nd2	2	0.52	1532.5	6.06	286.94	179	456
14_crop roi.nd2	2	0.47	1029.75	6.47	283.24	152	589
14_crop roi.nd2	1	0.48	1029.75	6.64	274.42	194	374
16_crop roi.nd2	2	0.65	2790.27	6.67	190.85	127	272
15_crop roi.nd2	4	0.51	1529.35	6.67	290.19	163	476
14_crop roi.nd2	3	0.48	1029.75	6.98	239.51	152	467
9_crop roi.nd2	3	0.61	1007.95	6.98	351.67	190	541
16_crop roi.nd2	4	0.62	2790.27	7.09	217.84	156	315
30_crop roi.nd2	1	0.78	1530.2	7.1	262.79	169	400
16_crop roi.nd2	1	0.75	2790.27	7.23	155.05	114	246
28_crop roi.nd2	1	0.53	783.99	7.35	349.97	211	498
9_crop roi.nd2	2	0.59	1007.95	7.35	224.99	130	411
22_crop roi.nd2	1	0.71	1637.37	7.93	296.39	169	501

29_crop roi.nd2	1	0.74	801.68	8.21	277.13	160	552
23_crop roi.nd2	1	0.62	854.18	8.22	320.07	213	521
28_crop roi.nd2	2	0.79	783.99	8.33	266.96	171	521
20_crop roi.nd2	2	0.81	1340.85	8.53	348.71	227	517
9_crop roi.nd2	1	0.72	1007.95	9.34	292.31	154	506
32_crop roi.nd2	1	1.09	2065.5	10.09	163.66	112	254
15_crop roi.nd2	2	0.85	1529.35	10.61	333.64	200	538
25_crop roi.nd2	1	1.08	1688.98	11.18	198.68	123	391
18_crop roi.nd2	2	1.08	2357.81	11.21	198.92	128	413
22_crop roi.nd2	2	0.9	1637.37	11.36	268	130	443
25_crop roi.nd2	2	1.18	1688.98	12.09	198.45	132	320
32_crop roi.nd2	2	1.66	2065.5	12.57	154.43	114	236
20_crop roi.nd2	1	1.11	1340.85	15.82	272.55	189	418
18_crop roi.nd2	1	2.4	2357.81	25.85	196.34	107	496

**Unsynapsed mean**	269.28
**Synapsed mean**	292.11

	**Mean ASY1 intensity - bead corrected**					
	Unsynapsed axial elements	Synapsed lateral elements	sections/samples (total)	Number of cells	Bead mean intensity	Number of beads
**pch2-1**	269.28	292.11	22	16	690.42	11

pch2 axis vs SC p=0.251 2 tail, paired t test

pch2-1 microspheres

Source	RoiID	ROIArea	MeasuredArea	Perimeter	MeanIntensity	MinIntensity	MaxIntensity
4.nd2	2	23.93	5452.92	17.28	563.81	417	911
6.nd2	1	24.18	5452.92	17.48	718.29	552	1341
6.nd2	2	24.18	5452.92	17.48	727.19	558	1450
6.nd2	1	24.18	5452.92	17.48	718.29	552	1341
6.nd2	2	24.18	5452.92	17.48	727.19	558	1450
6.nd2	3	24.18	5452.92	17.48	773.55	588	1338
6.nd2	4	24.18	5452.92	17.48	697.85	536	1193
6.nd2	5	24.18	5452.92	17.48	771.89	573	1421
6.nd2	6	24.18	5452.92	17.48	710.84	539	1454
1.nd2	1	26.01	278.87	18.12	585.2	400	1115
2.nd2	1	29.72	241.03	19.3	600.47	411	1149

**Mean intensity**	690.42
